# The melanoma tumor glyco-code impacts human dendritic cells’ functionality and dictates clinical outcomes

**DOI:** 10.3389/fimmu.2023.1120434

**Published:** 2023-02-20

**Authors:** Eleonora Sosa Cuevas, Benoît Roubinet, Stephane Mouret, Michel Thépaut, Florence de Fraipont, Julie Charles, Franck Fieschi, Ludovic Landemarre, Laurence Chaperot, Caroline Aspord

**Affiliations:** ^1^ Institute for Advanced Biosciences, Team: Epigenetics, Immunity, Metabolism, Cell Signaling and Cancer, Inserm U 1209, CNRS UMR 5309, Université Grenoble Alpes, Grenoble, France; ^2^ Etablissement Français du Sang Auvergne-Rhône-Alpes, R&D Laboratory, Grenoble, France; ^3^ GLYcoDiag, Orléans, France; ^4^ Dermatology, Allergology and Photobiology Department, CHU Grenoble Alpes, Grenoble, France; ^5^ Université Grenoble Alpes, CNRS, CEA, Institut de Biologie Structurale, Grenoble, France; ^6^ Medical Unit of Molecular Genetic (Hereditary Diseases and Oncology), Grenoble University Hospital, Grenoble, France; ^7^ Institut Universitaire de France (IUF), Paris, France

**Keywords:** glycan, lectin, human DC subsets, melanoma, immune subversion

## Abstract

Subversion of immunity is a hallmark of cancer development. Dendritic cells (DCs) are strategic immune cells triggering anti-tumor immune responses, but tumor cells exploit their versatility to subvert their functions. Tumor cells harbor unusual glycosylation patterns, which can be sensed through glycan-binding receptors (lectins) expressed by immune cells that are crucial for DCs to shape and orientate antitumor immunity. Yet, the global tumor glyco-code and its impact on immunity has not been explored in melanoma. To decrypt the potential link between aberrant glycosylation patterns and immune evasion in melanoma, we investigated the melanoma tumor glyco-code through the GLYcoPROFILE™ methodology (lectin arrays), and depicted its impact on patients’ clinical outcome and DC subsets’ functionality. Specific glycan patterns correlated with clinical outcome of melanoma patients, GlcNAc, NeuAc, TF-Ag and Fuc motifs being associated with poor outcome, whereas Man and Glc residues elicited better survival. Strikingly, tumor cells differentially impacting cytokine production by DCs harbored distinct glyco-profiles. GlcNAc exhibited a negative influence on cDC2s, whereas Fuc and Gal displayed inhibitory impacts on cDC1s and pDCs. We further identified potential booster glycans for cDC1s and pDCs. Targeting specific glycans on melanoma tumor cells restored DCs’ functionality. The tumor glyco-code was also linked to the nature of the immune infiltrate. This study unveils the impact of melanoma glycan patterns on immunity, and paves the way for innovative therapeutic options. Glycans/lectins interactions arise as promising immune checkpoints to rescue DCs from tumor’ hijacking to reshape antitumor immunity and inhibit immunosuppressive circuits triggered by aberrant tumor glycosylation.

## 1 Introduction

Immune evasion is crucial for tumor development (1). Unveiling complex interactions between tumor and immune cells is a challenging step to reshape proper antitumor immunity and achieve clinical success. Among immune cells enrolled in the tumor microenvironment, dendritic cells (DCs) are strategic immune cells that connect innate and adaptive immunity, and trigger and shape subsequent anti-tumor immune responses (2). DCs comprise several specialized subsets, among which conventional DCs type 1 (CD141/BDCA3^+^ cDC1s) and 2 (CD1c/BDCA1^+^ cDC2s), and plasmacytoid DCs (CD303/BDCA2^+^ pDCs) (3, 4). cDC1s are the main producers of type III IFN after Toll-like receptor (TLR)-3 signaling, possess a high cross-presentation capacity through CLEC9A and induce efficient CD8^+^ cytotoxic T cell responses (5, 6). cDC2s are specialized in the production of IL-12p70 after TLR4 or TLR8 stimulation and induce CD4^+^ T-cell responses (7). pDCs are the major producers of type I IFN after TLR7 or TLR9 stimulation, and are important for anti-viral immunity but also anti-tumor responses *via* their pleiotropic immunomodulatory functions (8).

Due to their unique ability to uptake antigens, perform cross-presentation, and activate antigen-specific adaptive immunity, DCs drive antitumor responses (2). However, tumor exploit DCs’ plasticity to escape immunity (9, 10). Such subversion has been documented in melanoma (10), where cDC2s (11) and pDCs (12, 13) display altered features, trigger pro-tumoral regulatory responses, exhibit altered cross-talk with anti-tumor immune effector cells (12, 14), and have been associated with bad clinical outcomes. Yet, the bases for such DCs’ hijacking remains elusive.

Aberrant glycosylation is considered a hallmark of cancer (15). Indeed, tumor cells harbor unusual glycosylation patterns on cell surface glycoproteins and glycolipids following deregulation of glycan biosynthesis, which influence interactions between tumor cells and the surrounding microenvironment (16–19). Melanoma tumor cells, which arise from malignant transformation of melanocytes, express glycoproteins (such as GP100, GPNMB) and glycolipids (such as GM3, GD3). Interestingly, it has been documented that melanoma tumor cells exhibit a high ganglioside diversity (20), display alterations in the glycosylation pattern of glycoproteins and glycolipids (21–23), and major perturbations of the expression of enzymes involved in glycosylation/deglycosylation processes (glycosyl-transferases, glycosidases) (21, 24, 25). Despite these specific alterations, the global glyco-code of melanoma cells remains unknown.

Abnormal glycosylation has been shown to play a critical role in cell proliferation, adhesion, migration, invasion and angiogenesis in many cancer types (18) including melanoma (23), thus promoting tumor progression. Emerging evidence suggest in few cancer types an immunosuppressive role of altered tumor glycosylation on innate immune responses through modulation of immune cells’ function (17, 26). Yet, the impact of altered glycan patterns on immunity remains undetermined in melanoma.

Strikingly, altered carbohydrate patterns on tumor cells can be recognized by glycan-binding receptors, named C-type lectin receptors (CLR). Immune cells especially DCs express an array of CLRs, allowing them to sense changes in glycan signature of their environment, and subsequently trigger immune responses. Upon binding to carbohydrate structures (glycans) through carbohydrate-recognition domains (CRDs), CLRs subsequently trigger intra-cellular signaling pathways through either immunoreceptor tyrosine-based activation motif (ITAM), immunoreceptor tyrosine-based inhibitory motif (ITIM), or by recruiting adaptors (27, 28). The ensuing activation of kinases such as SYK or recruitment of tyrosine phosphatases mainly SHP-1/2 leads to positive or negative modulation of antigen uptake, co-stimulatory molecules expression and cytokine production by DCs, thereby fine-tuning adaptive immune responses. Thus, CLRs are pivotal in the shaping of immune responses (29, 30), translating a variety of glycan structures into multiple effects ranging from immune suppression to potent immune activation (31).

Recognition of tumor glycans by CLRs is crucial for DCs to shape antitumor immunity, and decisive in the response’s orientation. The aberrant glycosylation of melanoma tumor cells could alter DCs’ features through CLR signaling and subsequently subvert anti-tumor immune responses. We recently highlighted that the CLR profiles on circulating and tumor-infiltrating DC subsets displayed strong perturbations in melanoma patients, correlated with unique DCs’ activation status and functionality, and dictated clinical outcomes (32). Furthermore, melanoma tumor cells directly altered CLR expression profiles of healthy DC subsets, suggesting that they displayed and released glycan motifs that may interact with DCs through CLR molecules. Melanoma tumors may shape DCs’ features by exploiting the plasticity of the CLR machinery. Yet the status of the tumor glyco-code together with its impact on DCs’ functionality has not been explored in melanoma. Knowing the importance of DC subsets in the response to immunotherapy as attested by several studies in the field (33–35), it is of major importance to decipher the glycan/DC axis in melanoma.

To decrypt the potential link between aberrant glycosylation patterns and immune evasion in melanoma, we aimed at characterizing the melanoma tumor glyco-code, and deciphering its impact on patients’ clinical outcome and DC subsets’ functionality. We depicted the whole tumor glyco-code of melanoma tumor cell lines through the GLYcoPROFILE™ methodology (lectin arrays), and established an *in-vitro* model of interaction between primary tumor cell lines (derived from melanoma patients) and purified DCs (derived from healthy donor blood) to depict the features of cDC2s, cDC1s and pDCs exposed to tumor cells displaying various glycan patterns. Our study revealed for the first time that the tumor glyco-code may dictate the clinical outcome of melanoma patients, and tumor cells harboring distinct glyco-profiles differently impacted DCs’ functionality. Thus, melanoma, through a specific glycan signature, may exploits CLR pathways to hijack DC subsets and escape from immune control. Our findings pave the way for the exploitation of this novel crucial immune checkpoint to design therapeutic strategies exploiting DCs’ potentiality while preventing hijacking by the tumor to reshape potent anti-tumor immunity.

## 2 Article types

Original Research Articles

## 3 Materials and methods

### 3.1 Melanoma patients and controls’ samples

This protocol was conformed to the French Blood Service’s (EFS-AuRA) Institutional Review Board and the ethics committee of Grenoble University Hospital (CHU-Grenoble) and declared under the reference #DC-2008-787. Written informed consent was obtained from all participants prior to their participation in this study. Blood samples were obtained from healthy donors (HD, n=20) and peripheral blood mononuclear cells (PBMCs) were isolated using Ficoll-Hypaque density gradient centrifugation (Eurobio). Lymph node or cutaneous metastatic tumors were obtained from 24 melanoma patients (naïve of treatment by immunotherapies) and reduced to cell suspensions by enzymatic digestion with 2 mg.ml-1 collagenase-D (Roche) 20 U.ml-1 DNase (Sigma) and mechanical disruption. The resulting cell suspensions were filtered and washed. Blood and tissue samples were biobanked and stored at -150˚C. Clinical features of melanoma patients are stated in **Supplementary Table 1**.

### 3.2 Cell culture of tumor cell lines derived from melanoma patients and healthy melanocytes

Clinical features of melanoma patients from which tumor cell lines were derived are stated in **Supplementary Table 1**. Tumor cell cultures (n=23) were grown in RPMI 1640 GLUTAMAX I supplemented with gentamicin (20µg/mL), non-essential amino acids (MEM 1X) (Invitrogen), sodium pyruvate (1mM) (Sigma) and 10% heat-inactivated FCS in a humidified incubator maintained at 37°C with 5% CO_2_ atmosphere. For each patient, the cell suspension resulting from tumor disruption was put into a culture flask. After 24h, the medium containing immune infiltrate was removed and adherent tumor cells were further cultured. Tumor cells were checked for Mycoplasma contamination using the MycoAlert PLUS detection kit (Lonza) and tumor cell lines with less than ten passages were used for the experiments. Human adult melanocytes (n=6) were cultured following manufacturer’s instructions (LONZA).

### 3.3 GLYcoPROFILE™ of tumor cell lines derived from melanoma patients and healthy melanocytes

The glyco-code of melanoma tumor cells was assessed by performing the GLYcoPROFILE™ with the LEctPROFILE^®^ plates from GLYcoDiag (Orléans, France). The assessment of interactions of lectins with glycans on cell surfaces were achieved according to GLYcoDiag’s protocol (36, 37). When cells grew up to 80–90% confluence in 75 cm^2^ culture flask, cells were washed with PBS and harvested with a Trypsin/EDTA solution. After washing and centrifugation, the cells were suspended in PBS and labeled with carboxyfluorescein diacetate succinimidylester (CFDA-SE, Sigma-Aldrich, St. Louis, MO, USA) in PBS. Next, 100 μL of labeled cells (about 2 × 10^5^ cells) were added in each well of the LEctPROFILE^®^ plates and incubated 2 h at room temperature under gentle agitation. After washing with PBS, fluorescence intensity was measured using a microplate reader (λ_ex_ = 485 nm, λ_em_ = 530 nm, Fluostar OPTIMA, BMG LABTECH, France). In parallel, a calibration curve was achieved with the labeled cells solution to determine the number of cells stayed in interactions with lectins.

### 3.4 Co-culture of tumor cells derived from patients with panDCs derived from healthy donors

Tumor cell cultures were grown in RPMI 1640 GLUTAMAX I supplemented with gentamicin (20µg/mL), non-essential amino acids (MEM 1X) (Invitrogen), sodium pyruvate (1mM) (Sigma) and 10% heat-inactivated FCS in a humidified incubator maintained at 37°C with 5% CO_2_ atmosphere. The medium was changed every other day and the cells were cultured until 70-80% confluence when they were used in the experiments. PanDCs (containing a mix of cDC2s, cDC1s and pDCs) were purified from frozen PBMCs derived from healthy donors using the EasySepTM human panDCs pre-enrichment kit (StemCell). For co-culture experiments, tumor cells were trypsinated using Trypsin/EDTA (StemCell), washed, and seeded in 48-well flat bottom plates. Purified human panDCs were co-cultured with or without confluent tumor cells at 1.10^6^/ml in 48-well flat bottom plates for 20 hours at 37°C with 5% CO_2_. In some experiments, to block surface tumor glycans, soluble lectins (GLYcoDiag) at 27.8 µg.mL^-1^ were added or not to tumor cell cultures for 2 hours at 37°C with 5% CO_2_ and washed, prior to co-culture with panDCs.

### 3.5 Assessment of cytokine production by panDCs (cultured with tumor cells or tumor-derived supernatants) upon TLR triggering

After 20 hours of co-culture with or without either tumor cells (500.000 PanDCs on a confluent layer of 50.000 to 125.000 tumor cells depending on the cell line) or tumor-derived supernatants (50% of total medium), panDCs were collected in 96-well U-bottom plates and cultured at 1.10^6^/mL for 5 hours with or without TLR ligands, including polyinosinic-polycytidylic acid (polyI:C, 30 µg.mL^-1^), Resiquimod (R848, 1 µg.mL^-1^) and Class-A CpG oligonucleotide ODN-2336 (CpG_A_, 1µM) (*In vivo*gen). 1 µg.mL^-1^ of Brefeldin A (BD) was added after 1 hour. Afterwards, cells were stained for surface markers to depict DC subsets (CD11c, HLA-DR (BD), Lin, CD45 (Biolegend), CD1c/BDCA1 (Beckman), CD3003/BDCA2 and CD141/BDCA3 (Miltenyi)) and Live and Dead staining (Invitrogen) was used to exclude dead cells. Thus, DC populations were identified as alived singlet CD45^+^HLA-DR^+^Lin^-^ cells and subdivided as CD11c^+^CD1c^+^ cDC2s, CD11c^+^CD141^+^ cDC1s and CD11c^-^CD303^+^ pDCs. Samples were then fixed and permeabilized according to the manufacturer’s instructions (BD Cytofix/Cytoperm™ Plus kit) and intracellular cytokine staining was performed using fluorochrome-labeled anti-human antibodies (TNFα, IL-12p40/70 (BD), IFNα (Miltenyi), and IFNλ1 antibody (Novus) stained with mix-n-stain CF488 (Biotum). Analyses were done by flow cytometry using LSRII Flow Cytometer and FACSDIVA software v.9 (BD). Isotype controls were used to differentiate positive cells from nonspecific background staining (CD45^+^ cells also served to determine the threshold of positivity). Proportions of cytokine-producing cells were analyzed. To ensure quality control during the study, we performed a standardization of the fluorescence intensities using cytometer setup and tracking beads (CST) (BD).

### 3.6 Analysis of tumor-derived factors by Luminex

Melanoma tumor cell lines were cultured in control conditions or incubated with soluble lectins (HPA, WGA, MAA) for 2h and washed. Supernatants were harvested after 20h of culture and IL1β, IL6, IL8, IL10, MCP1, MIP1α, MIP1β and TGFβ secretions were measured by LUMINEX technology using MAGPIX^®^200 Instrument with xPONENT^®^ software (Bio-Rad, Cressier).

### 3.7 Evaluation of the tumor immune infiltrate by flow cytometry

We analyzed the nature and density of immune infiltrate in the samples from which tumor cells were derived. Frozen samples were thawed and stained in PBS 2% fetal calf serum (FCS) with several fluorochrome-labeled anti-human antibodies. The combination of the following surface markers allowed to define CD45, CD3 T cells, CD8 T cells, cDC1s, cDC2s and pDCs: CD45, CD3, CD8, CD11c, HLA-DR (BD Biosciences, Le Pont de Claix), Lin (Biolegend, Paris), CD45, CD1c (Beckman, Roissy), CD303, and CD141 (Miltenyi, Paris). Stained cells were then analyzed using LSRII Flow Cytometer and FACSDiva software v.8 (BD).

### 3.8 Statistical analyses

Statistical analyses were performed using Mann-Whitney non parametric U-test, Wilcoxon matched-paired signed rank test, both matched two-way repeated measures ANOVA or mixed-effects model (REML) with Bonferroni’s multiple comparisons test, and Spearman correlations using Graph Pad Prism software. Global lectin data were shown as medians and individual tumor cell line GLYcoPROFILE™ data was expressed as mean ± SD of two independent experiments. Significance threshold was placed at p-value < 0.05. Survival analyses (Kaplan-Meier), correlations, heatmaps and Principal Component Analysis (PCA) were performed using the survival, dplyr, corrplot, gplots, ggbiplot, RColorBrewer, MissMDA and FactoMineR packages using RStudio software R version 4.2.1. Specifically, heatmaps were done with function heatmap.2 from the gplots package, and clustering were done using the hclust function, which performs hierarchical clustering.

### 3.9 Data availability

The data generated in this study are available upon request from the corresponding author.

## 4 Results

### 4.1 Melanoma tumor cells differentially impact cytokine production by DC subsets upon TLR triggering

**Figure 1:**
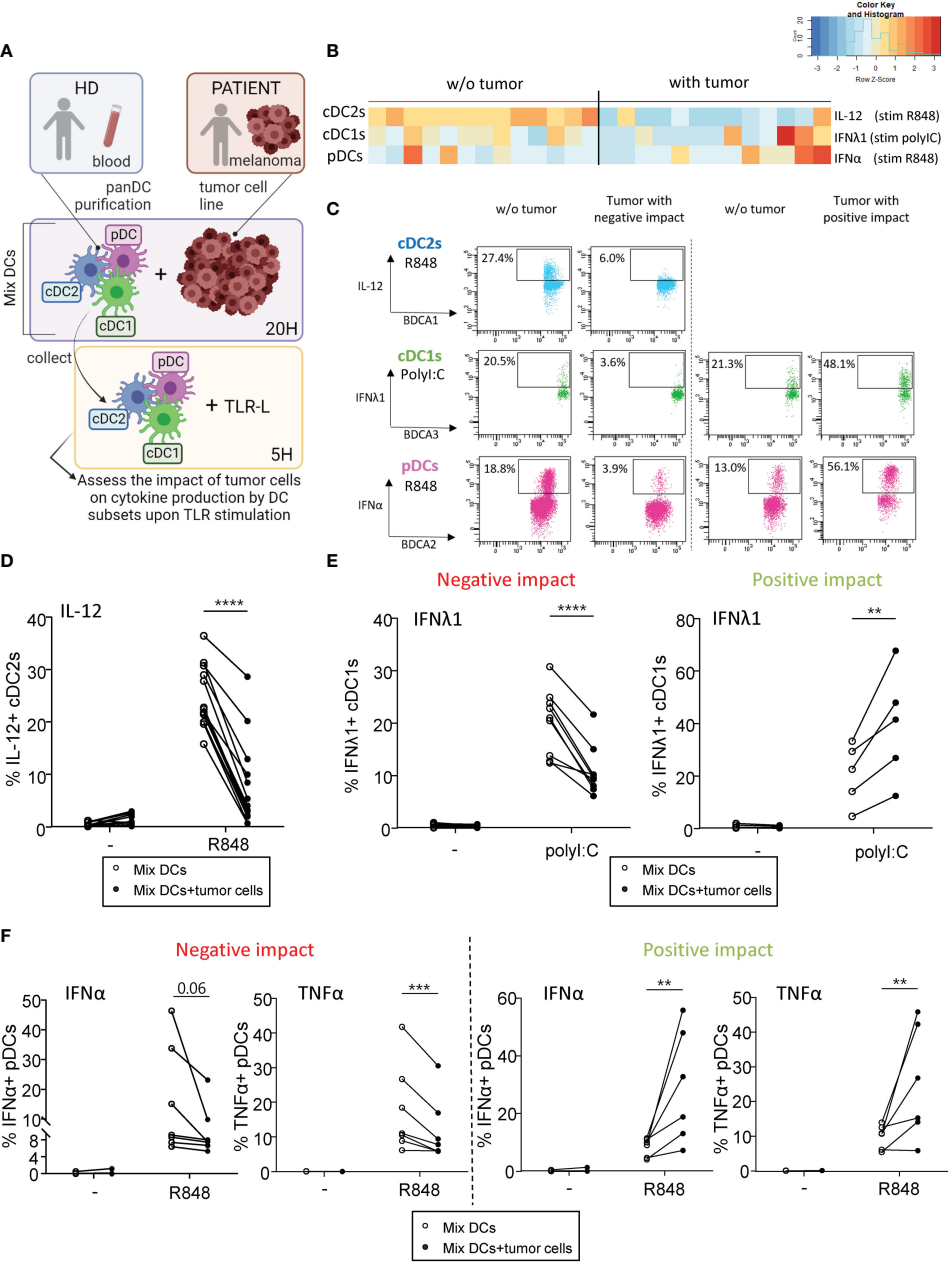
Primary tumor cell lines derived from melanoma patients differentially influenced cytokine production by DC subsets upon TLR triggering PanDCs (mixture of the three DC subsets cDC2s, cDC1s, pDCs) were purified from several HD blood and co-cultured with distinct primary tumor cell lines (derived from melanoma patients) for 20 hours. Collected panDCs were stimulated for 5 hours with or without TLR-L (polyI:C or R848) and the production of cytokines was assessed by intracellular cytokine staining using flow cytometry. **(A)** Schematic representation of the experimental layout to investigate the impact of distinct tumor cell lines on cytokine production by healthy DC subsets. **(B)** Heat map based on cytokine production by cDC2s (IL-12p40/p70), cDC1s (IFNλ1) and pDCs (IFNα) upon TLR stimulation after culture of PanDCs with distinct tumor cell lines (n = 13 tumors). Each tumor cell line was co-cultured with one to four PanDCs mixtures purified from different HD blood (n=13 donors) and mean percentages of cytokine^+^ DCs were calculated and shown in the heat map. **(C)** Representative dot plots highlighting diverse impacts of co-culture of PanDCs with tumor cell lines derived from melanoma patients on cytokine production by DC subsets upon TLR stimulation (PolyI:C or R848) (“negative” or “positive” impact). Dot plots are pre-gated on CD45^+^Lin-HLA^-^DR^+^ cells, in addition to CD11c^+^CD1c/BDCA1^+^ for cDC2s, CD11c^+^CD141/BDCA3^+^ for cDC1s, and CD11c^-^CD303/BDCA2^+^ for pDCs as illustrated in **Supplementary Figure 1**. **(D–F)** Frequencies of cytokine-expressing cDC2s **(D)**, cDC1s **(E)** and pDCs **(F)** upon TLR triggering after co-culture with (filled circles) or without (open circles) tumor cell lines derived from melanoma patients (n = 13 tumors). For cDC1s and pDCs, groups were separated depending of the “negative” or “positive” impact of the tumor on IFNλ1 or IFNα production respectively. Results are expressed as percentages of cytokine-expressing cells within the corresponding DC subset. Each point represents a different tumor cell line and illustrates the mean of co-cultures experiments of each cell line with one to four different HDs (n=34 PanDCs/tumor co-cultures in total). Only significant statistics are shown on graphs. *P*-values were calculated using matched two-way repeated measures ANOVA with Bonferroni’s multiple comparisons test. **P-value ≤ 0.01, ***P-value ≤ 0.001, ****P-value ≤ 0.0001.

Given that interactions between aberrant glycans on tumor cells and CLRs on DCs could potentially contribute to immune subversion, we first investigated the impact of different primary tumor cell lines derived from melanoma patients on cytokine production by DC subsets upon TLR stimulation. PanDCs (mixture of cDC2s, cDC1s and pDCs) were purified from healthy donors’ (HD) blood and co-cultured for 20 hours with different primary tumor cell lines derived from melanoma patients (n=14 lines). Using a multi-parametric flow cytometry approach (**Supplementary Figure S1**), we simultaneously depicted the three major DC subsets in circulation. Thus, among alive CD45^+^Lin^-^HLA-DR^+^ cells, cDC2s, cDC1s and pDCs were defined as respectively CD11c^+^CD1c/BDCA1^+^ cells, CD11c^+^CD141/BDCA3^+^ cells and CD11c^-^CD303/BDCA2^+^ cells. After co-culture or not with tumor cells, cytokine production by DC subsets was assessed upon TLR stimulation (PolyI:C or R848) (**Figure 1A**). To illustrate differences between “Mix DCs” and “Mix DCs+tumor cells” conditions, we performed supervised nonhierarchical clustering. The resulting heat map reveals heterogeneity in the impact of tumor cells on cytokine production by DC subsets (**Figure 1B**). All tumors have a “negative” impact on cDC2s’ cytokine production, whereas “positive” or “negative” impacts were seen on cDC1s and pDCs depending on the cell lines (**Figure 1C**). Indeed, all tumor cell lines derived from melanoma patients abrogated IL-12p40/p70 and TNFα production by cDC2s upon R848 (**Figures 1D**, **S2A**). To classify cell lines based on their positive or negative impact on cDC1s and pDCs in an unbiased manner, we performed an unsupervised hierarchical clustering of the cell lines, based on the fold change in cytokine production between conditions with and without tumor cells(**Supplementary Figure S2B**). Such analysis was done for each DC subset independently, based on IFNλ1 and TNFα production for cDC1s, and on IFNα and TNFα production for pDCs. This analysis allows separating the cell lines in two groups displaying either positive or negative impact on cDC1s or pDCs. Interestingly, IFNλ1 production by cDC1s upon PolyI:C significantly decreased (**Figure 1E**, left panel) or increased (**Figure 1E**, right panel) compared to controls depending on the cell lines. Yet, TNFα production by cDC1s upon PolyI:C was unaffected by tumor cells, even though we observed a trend of tumors with a positive impact to increase TNFα production in a TLR independent manner (**Supplementary Figure S2C**). Moreover for pDCs, tumors either altered cytokine production or boosted frequencies of IFNα^+^ or TNFα^+^ pDCs after TLR stimulation (**Figure 1F**). Importantly, similar impacts were observed for each tumor on different donors of DCs, indicating a tumor-based effect (**Supplementary Figure S2D**). In addition, tumor cells do not affect cytokine production by DCs in absence of TLR-L, as illustrated by the absence of changes in control condition (-) (**Figures 1D–F**, **S2A, C**). To assess whether the positive or negative impact of tumor cell lines on DCs could be mediated by tumor-derived conditioned medium, we analyzed the functionality of DCs cocultured either with tumor cells or with tumor-derived supernatants. For positive tumor lines, the impact on cDC1s and pDCs was not mediated by tumor-derived supernatants, whereas for negative tumor lines, the impact on cDC2s and pDCs (but not on cDC1s) was partially recapitulated by tumor-conditioned medium (**Supplementary Figures 3A, B**). We further investigated the factors secreted by tumors cells that may influence DCs’ activation or functionality, and compare their quantity between tumor cell lines displaying positive and negative impacts on DCs (**Supplementary Figure 3C**). All tumor cells secreted high levels of IL6, IL8, MCP1 and TGFβ, and lines with positive impact on DCs had lower levels of IL6 and MCP1 compared to lines with negative impact on DCs. Altogether, these findings allowed us to distinguish tumors with positive impact from those with negative effect on cDC1s’ and pDCs’ functionality. Thus, tumors derived from melanoma patients differentially impact cytokine production by DC subsets upon TLR triggering.

### 4.2 Melanoma tumor cells harbor different glyco-code compared to healthy melanocytes

**Figure 2:**
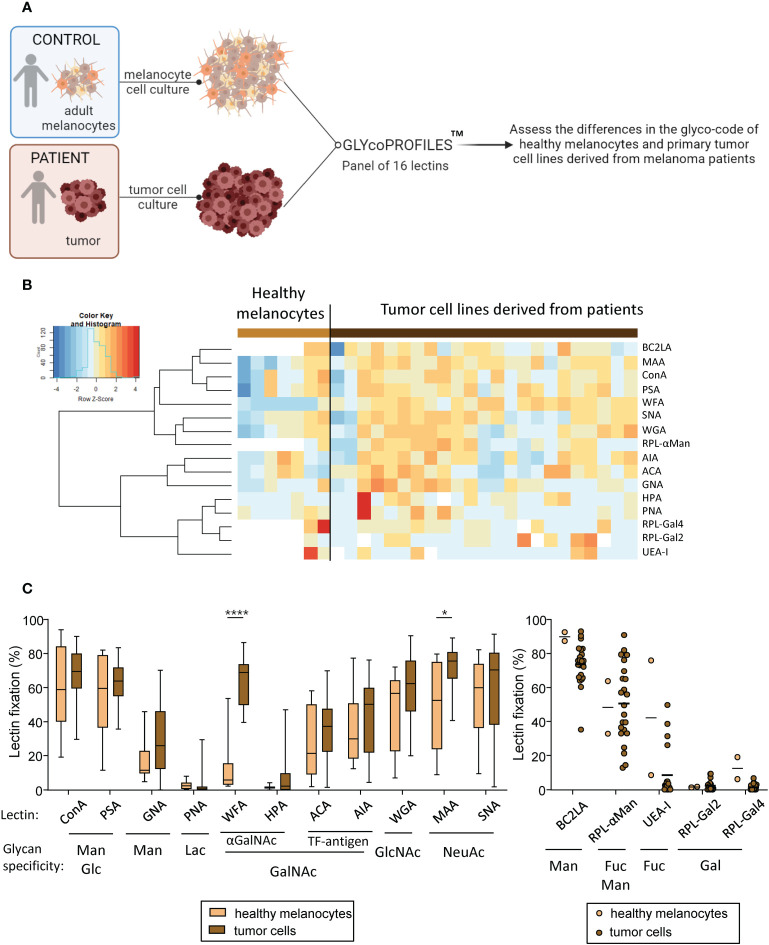
Primary tumor cells derived from melanoma patients display differences in their glyco-code when compared to healthy melanocytes. GLYcoPROFILE™ (lectin arrays from GLYcoDiag) were performed on human adult melanocytes and tumor cell lines derived from melanoma patients. **(A)** Schematic representation of the experimental layout to depict the glyco-code of adult melanocytes and tumor cell lines derived from melanoma patients. **(B)** Heat map based on frequencies of 16 different lectins interaction (binding different glycans) on healthy melanocytes (n = 2 to 6; depending on the lectin studied) and tumor cell lines derived from melanoma patients (n = 23). **(C)** Levels of lectin interaction (indicators of levels of glycan expression) of ConA, PSA, GNA, PNA, WFA, HPA, ACA, AIA, WGA, MAA and SNA (left panel) by healthy melanocytes (light brown; n = 6) and tumor cells (dark brown; n =23). Results are expressed as percentages of lectin binding within each group. Interleaved box and whiskers representation plotting from minimum to maximum. Only significant statistics are shown on the graphs. *P*-values were calculated using two-way ANOVA with Bonferroni’s multiple comparisons test. *P-value ≤ 0.05, ****P-value ≤ 0.0001. The levels of lectin fixation of BC2LA, RPL-αMan, UEA-I, RPL-Gal2 and RPL-Gal4 (right panel) on healthy melanocytes (n=2) and tumor cell lines (n=23) are illustrated using a scatter dot plot representation.

These previous results prompted us to investigate the potential differences between tumor cell lines that could explain the differential impacts observed on the immune system. We recently highlighted that melanoma tumor cells triggered perturbation in the CLR expression profile of DCs. To explore the potential link between glycosylation patterns on tumor cells (CLR-ligands) and immune modulations, we assessed the tumor glyco-code by performing lectin arrays (GLYcoPROFILE™ technology) of primary tumor cell lines derived from melanoma patients (n=23) using human adult melanocytes as control (n=2 to 6) (**Figure 2A**). Lectin fixation indicates expression of the corresponding glycan motifs on tumor cells. The complete panel of lectins studied (n=16) together with their glycan specificities are shown in **Supplementary Table 2**. To illustrate the potential differences in lectin recognition between healthy melanocytes and primary melanoma tumor cells, we performed Euclidean distance-based hierarchical clustering (**Figure 2B**). Given the low number of samples (n = 2) for the control group regarding the fixation of BC2LA, RPL-αMan, UEA-I, RPL-Gal2 and RPL-Gal4 lectins, differences of fixation for these lectins between both groups could not be statistically interpreted (**Figure 2C**, right panel). Interestingly, when looking to other individual lectin fixation levels, we found higher levels of GalNAc and NeuAc residues (revealed by the interaction of WFA and MAA respectively) in melanoma tumor cell lines when compared to healthy melanocytes (**Figure 2C**, left panel). Given intra-group heterogeneity of lectin fixation by tumor cell lines derived from melanoma patients, we investigated whether the glyco-code depends on the initial tumor localization (cutaneous or lymph node metastasis) (**Supplementary Figure S4**). The results are illustrated on a heatmap generated by Euclidean distance-based clustering (**Supplementary Figure S4A**), and revealed higher levels of Man, Fuc and Gal residues (seen by GNA, UEA-I and RPL-Gal2 interaction respectively) in tumor cell lines derived from lymph node metastasis when compared to cutaneous metastasis (**Supplementary Figure S4B**). Thus, tumor cells derived from melanoma patients seem to harbor a specific glyco-code characterized by high levels of GalNAc and NeuAc residues compared to their healthy counterpart, with variations in the expression of Man, Fuc and Gal motifs depending on their tissue localization.

### 4.3 The glyco-code profiles on melanoma cells correlate with clinical outcome of the patients

**Figure 3:**
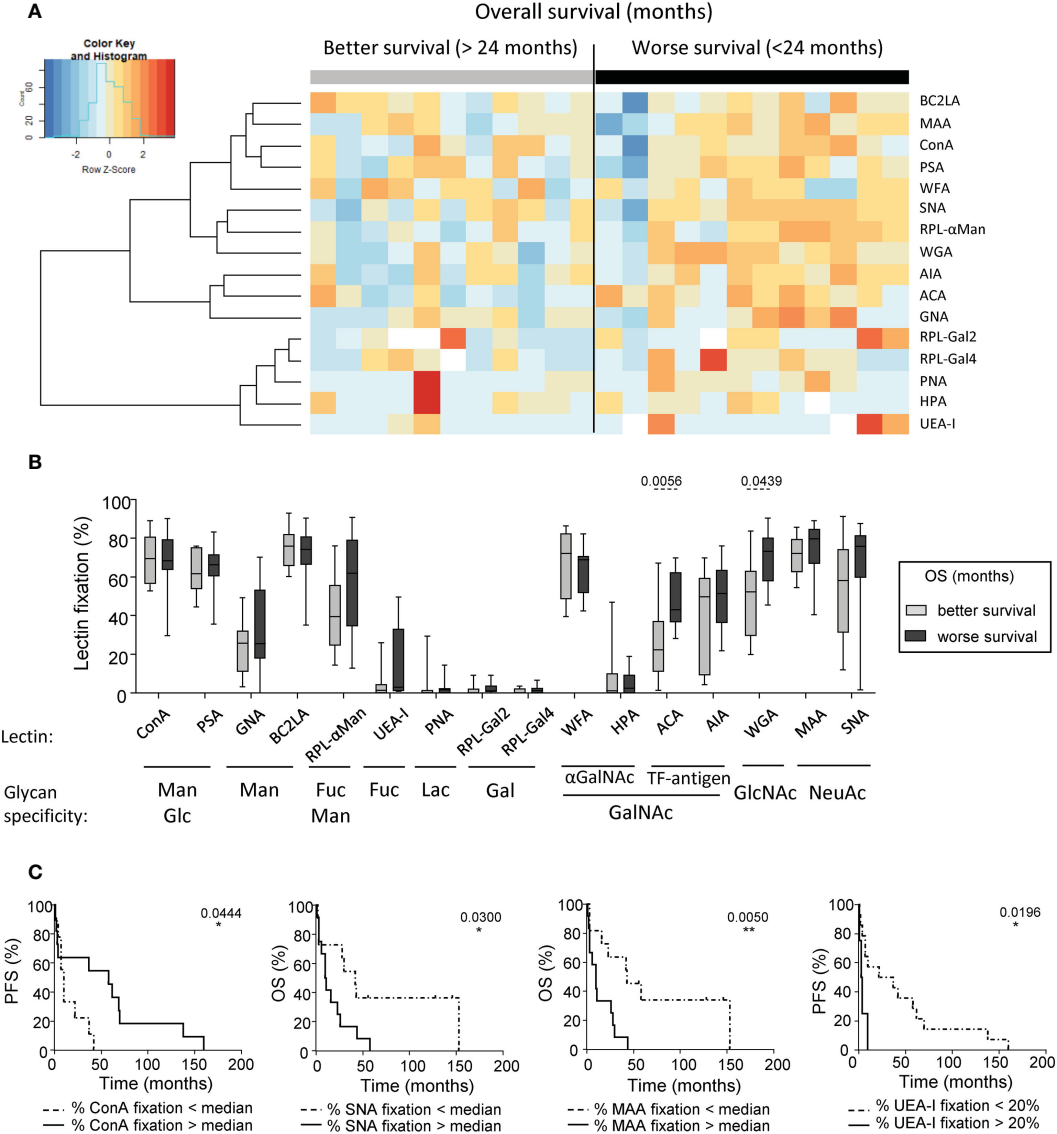
Differences of glycan expression in the tumor glyco-code were linked to the clinical outcome of melanoma patients. GLYcoPROFILE™ (lectin arrays from GLYcoDiag) were performed on tumor cell lines derived from melanoma patients (n = 23). Samples were then separated given patient’s clinical data. **(A)** Heat map based on frequencies of 16 different lectins fixation (binding different glycans) on tumor cell lines derived from patients with better (n =11) or worse (n = 12) overall survival (OS) from sampling time (separation based on the median OS of 24 months). **(B)** Frequencies of lectin fixation (indicators of levels of glycan expression) by tumor cells derived from patients with better (n = 9 to 11) or worse (n = 10 to 12) overall survival (from sampling time). Results are expressed as percentages of lectin binding within each group. Interleaved box and whiskers representation plotting from minimum to maximum. *P*-values were calculated using Mann-Whitney non-parametric U-test. **(C)** Comparative PFS (from diagnostic time) and OS (from sampling time) of patients with tumor cell lines displaying low or high fixation of ConA (n=9-11 patients/group), SNA (n=11-12 patients/group), MAA (n=11-12 patients/group), or UEA-I (n = 4-14 patients/group). Groups were separated using median percentage of fixation of ConA (69.48%), SNA (70.46%) or MAA (75.5%), and a threshold of 20% for UEA-I fixation. Comparison using Log-rank test. *P-value ≤ 0.05, **P-value ≤ 0.01.

To evaluate the clinical relevance of tumor glyco-code profiles of melanoma patients, we investigated differences between glyco-code profiles of tumor cell lines derived from patients with good or poor clinical outcome. The global view of tumor glyco-code profiles upon separation of patients based on better or worse overall survival (median OS) (**Figure 3A**) or progression-free survival (median PFS) (**Supplementary Figure S5A**) revealed a pattern of higher expression of Thomsen-Friedenreich antigen (TF-antigen), GlcNAc, Fuc and NeuAc residues (seen by the fixation of ACA, WGA, RPL-αMan, UEA-I, MAA and SNA) in tumor cell lines from patients with worse clinical outcome. Strikingly, tumor cells with higher levels of fixation of ACA and WGA (recognizing TF-antigen and GlcNAc residues respectively) were found in patients with worse OS, whereas tumor cells with higher levels of fixation of HPA (recognizing terminal αGalNAc) were found in patients with a better PFS (**Figures 3B**, **S5B**). By further studying the link between specific glycan motifs on melanoma tumor cells and the clinical outcome of patients through Kaplan-Meier analyses (**Figure 3C**, **Supplementary Table 3**), we highlight that higher levels of Man/Glc residues on tumor cell lines (seen by ConA fixation) correlate with better PFS (**Figure 3C**; left panel), whereas higher levels of NeuAc and Fuc residues (unveiled by SNA, MAA or UEA-I fixation respectively) predict a worse clinical outcome in melanoma patients (**Figure 3C**; right panels). Altogether, these observations demonstrate that specific glycan patterns on tumor cells correlate with clinical outcome of melanoma patients, especially GlcNAc, NeuAc, TF-antigen and Fuc motifs being associated with poor outcome, whereas Man and Glc residues elicit better survival.

### 4.4 Tumor cells displaying a “negative” impact on cDC1s and pDCs harbored a glyco-code enriched in Gal residues

**Figure 4:**
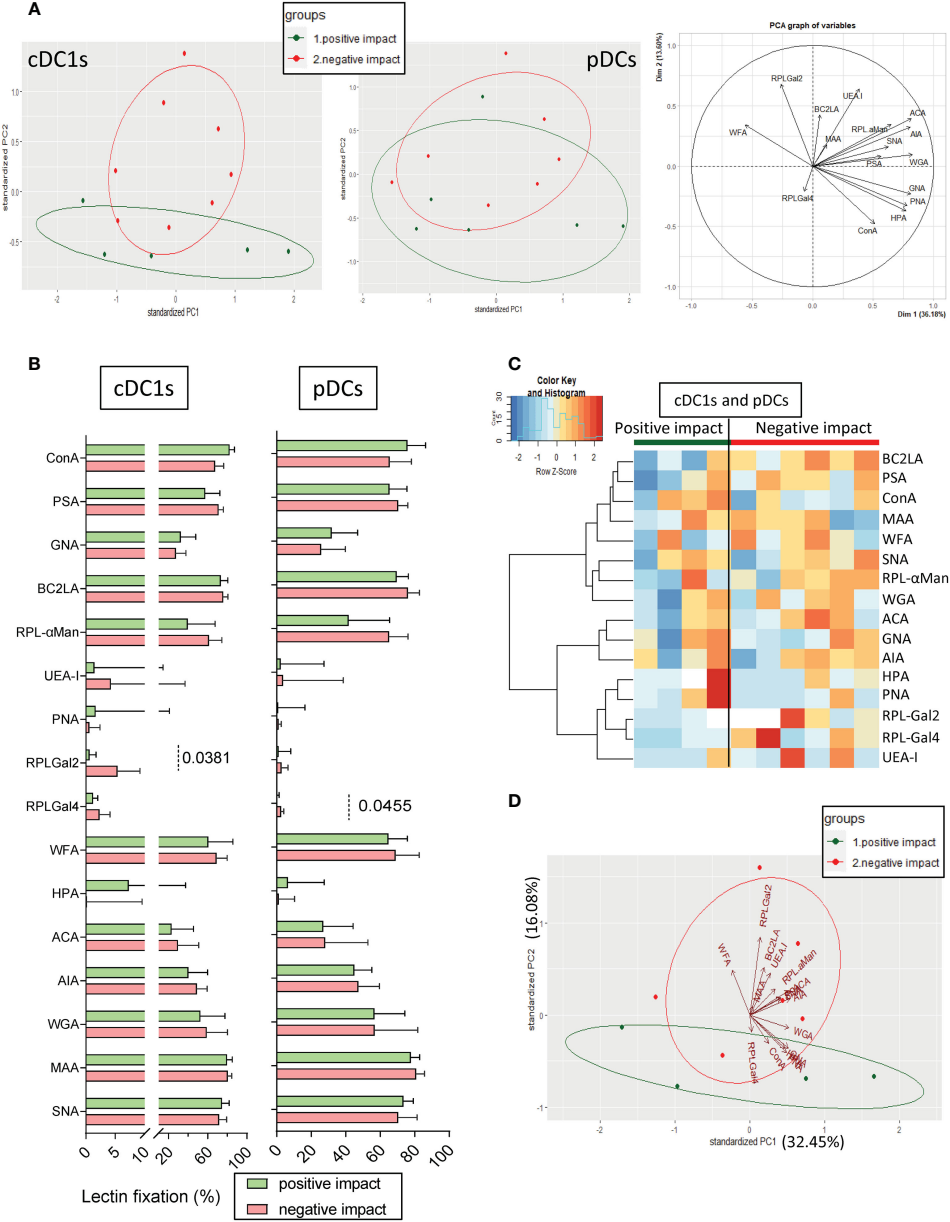
Tumor cell lines with positive or negative impacts on cDC1s and pDCs harbor differences in their glyco-code. Glycan expression by tumor cell lines derived from melanoma patients was assessed through lectin fixation (GLYcoPROFILE™). Samples were then grouped depending on their individual impact (positive or negative) on cDC1s and pDCs (groups defined in **Figure 1**). **(A)** Principal component analysis (PCA) based on lectin fixation of tumor cell lines derived from melanoma patients (including graph of variables; right panel). Tumor cells were separated given their positive or negative impact on cytokine production by cDC1s (left panel) and pDCs (middle panel) (n = 5 to 8 tumors per group). **(B)** Frequencies of lectin fixation (indicators of glycan expression levels) by tumor cells which positively or negatively impacted cytokine production by cDC1s or pDCs (n = 5 to 8 per group). Results are expressed as percentages of lectin binding within each group. Interleaved bars representation plotting median with interquartile range. Only significant statistics are shown on graphs. *P*-values were calculated using Mann-Whitney non parametric test. **(C)** Heat map based on frequencies of fixation of 16 different lectins (binding different glycans) on tumor cell lines that positively (n = 4) or negatively (n = 6) impacted cytokine production by cDC1s and pDCs. **(D)** PCA based on lectin fixation by tumor cells separated given their positive or negative impact on cDC1s and pDCs (n = 4 to 6 per group).

As we previously highlighted different impacts of tumor cells on cytokine production by cDC1s and pDCs upon TLR stimulation (**Figure 1**), we further explored the differences between the glyco-code profiles of tumor cells with a “negative” or “positive” impact on DC subsets’ functionality. The results are illustrated on a heatmap generated by Euclidean distance-based clustering (**Supplementary Figure S6A**) and we ran a PCA analysis (**Figure 4A**) to compare the glyco-code profiles of tumor cells exhibiting a “negative” and “positive” impact on cDC1s or pDCs. Strikingly, PCA based on tumor glyco-code allowed separating tumor cells that negatively or positively impacted cDC1s (**Figure 4A**, left panel). Differences between groups seemed to be mostly driven by RPL-Gal2 fixation by tumor cells (**Figure 4A**, graph of variables). Furthermore, when comparing levels of lectin fixation for both groups (negative or positive), tumor cell lines displaying a “negative” impact on cDC1s harbored higher levels of α-Gal residues (revealed through RPL-Gal2 fixation), and tumor cells negatively impacting pDCs exhibited higher levels of β-Gal residues (unveiled by RPL-Gal4 fixation) (**Figure 4B**), even though low. We further assessed whether tumor cells having the same effect simultaneously on cDC1s and pDCs, either negative (n= 6) or positive (n=4), would share common features regarding their glyco-code. We observed that tumor cell lines displaying a negative impact on both cDC1s and pDCs harbored higher levels of Gal residues (revealed by RPL-Gal2 and RPL-Gal4 fixation) when compared to tumor cells with a positive impact (**Figures 4C, D**, **S6B**). Thus, tumor cells displaying a “negative” impact on cDC1s and pDCs’ functionality exhibited higher levels of Gal residues at their surface.

### 4.5 Blocking specific lectins on tumor cells with a “positive” impact on cDC1s and pDCs boost their stimulatory effect on DCs upon TLR triggering

**Figure 5:**
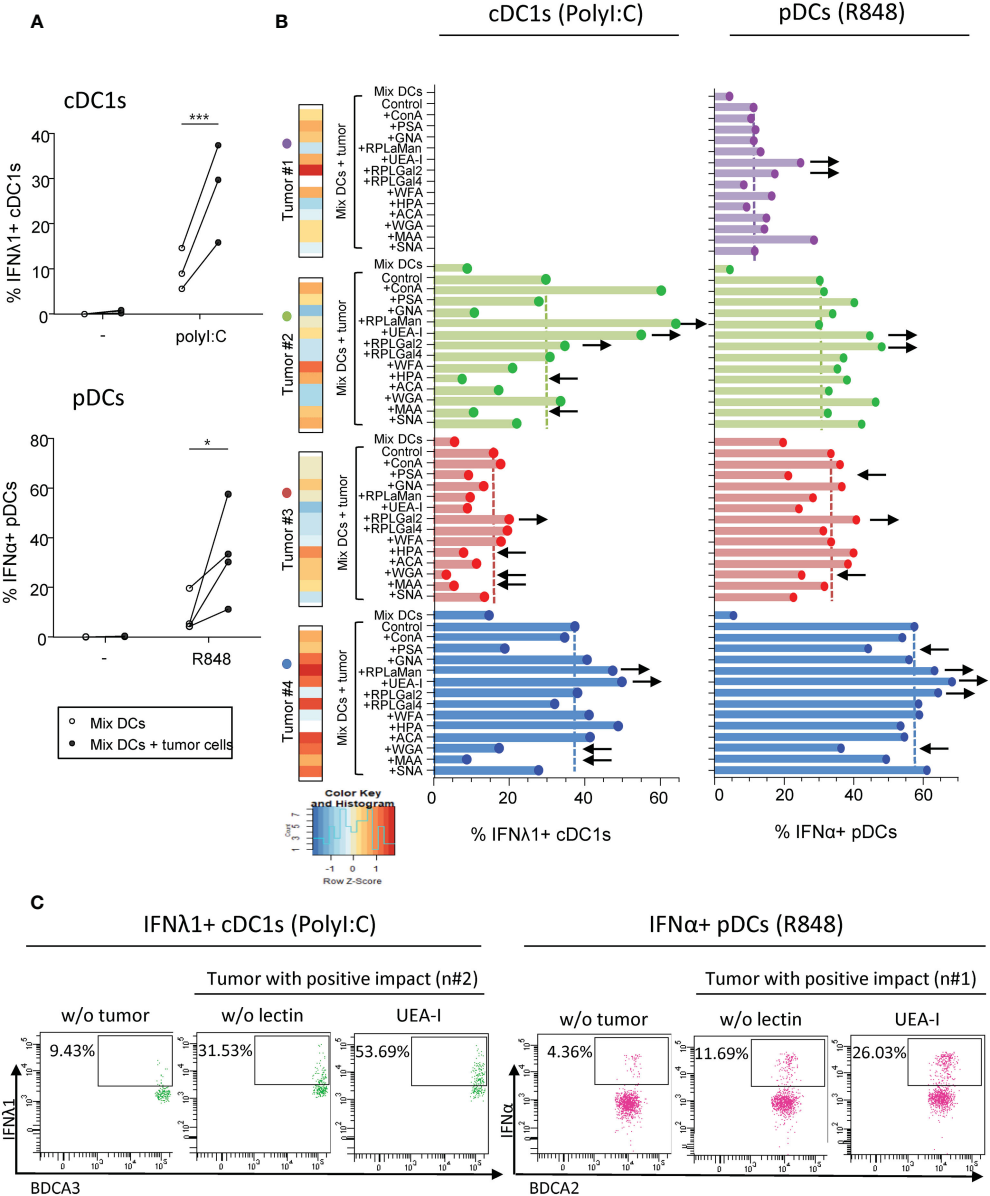
Pre-treatment of “positive” tumor cells with specific lectins *in-vitro* further boosts their good impact on cytokine production by cDC1s and pDCs. PanDCs were co-cultured for 20 hours with “positive” tumor cell lines previously cultured or not with soluble lectins for 2 hours. Collected panDCs were then stimulated for 5 hours with or without TLR-L (polyI:C, R848) and the production of cytokines was measured using flow cytometry. **(A)** Frequencies of IFNλ1^+^ cDC1s (higher panel) or IFNα^+^ pDCs (lower panel) upon TLR triggering after co-culture with (filled circles) or without (open circles) tumor cell lines that positively impacted cDC1s or pDCs’ functionality (called “positive” tumors) and that were previously untreated with soluble lectins (n = 3 to 4 different panDC/tumor combos per group). Results are expressed as percentages of cytokine-expressing cells within each group. Only significant statistics are shown on graphs. *P*-values were calculated using matched two-way repeated measures ANOVA with Bonferroni’s multiple comparisons test. *P-value ≤ 0.05, ***P-value ≤ 0.001. **(B)** Proportions of IFNλ1^+^ cDC1s (left panels) and IFNα^+^ pDCs (right panels) upon PolyI:C or R848 stimulation respectively after 20h of culture or not with “positive” tumors previously treated or not with soluble lectins (n = 3 or 4 tumors). Lectin binding by each tumor cell line (#1 to 4) was illustrated on the left part and color scaling was done per lectin. Black arrows indicate recurrent down- (←) or up (→) modulations of cytokine secretion compared to “MixDCs+tumor” condition. **(C)** Representative dot plots highlighting the boost in cytokine production (upon TLR stimulation) by cDC1s (left panels) and pDCs (right panels) after co-culture with “positive” tumor cell lines (#2 and #1 respectively) pre-treated with the lectin UEA-I.

To further decipher the role of specific glycan in modulating DCs’ functionality, we investigated whether pre-treating tumor cell lines with soluble lectins could alter their impact on DCs. We co-cultured purified “mix DCs” with tumor cells previously treated with single or mixture of soluble lectins. To avoid contact of DCs with lectins, tumor cells were washed to remove any potential remaining lectins. DCs were then stimulated using TLR-L (PolyI:C or R848), and cytokine production was assessed by intracellular cytokine staining. The comparison of cytokine production with and without lectins would allow us to decipher the involvement of specific glycans in triggering or inhibiting DCs’ functionality (**Supplementary Figure 7**). With an attempt to identify glycans positively influencing DCs’ function, we first chose tumor cell lines with a positive impact and assessed which lectin would abrogate its effect. This part of the study was limited to four tumor cells derived from melanoma patients (#1 to #4), among which three positively impacted cytokine production by cDC1s and four by pDCs after TLR stimulation (**Figures 5A**, **S8A**). We verified that these four “positive” tumor cells pre-treated with soluble lectins had no impact on cytokine production by cDC1 and pDCs in absence of TLR stimulation (condition “w/o stim”) (**Supplementary Figures S8B, C**). Notably, when tumor cells display high levels of HPA (#2 and #3), WGA (#3 and #4) or MAA (#2, #3 and #4) fixation, their pre-treatment with these soluble lectins inhibited their positive impact on cDC1s (**Figure 5B** left panels, **Supplementary Figure 9** left panels), suggesting that αGalNAc, GlcNAc and NeuAc residues positively impact cDC1s upon TLR triggering. In addition, pre-treatment of tumor cell lines with soluble PSA or WGA inhibited the positive impact on pDCs (tumor #3, #4) (**Figure 5B** right panels, **Supplementary Figure 9** right panels), suggesting that Man/Glc motifs favor IFNα production by pDCs. Interestingly, treatment of the “positive” tumor cells with HPA, WGA or MAA triggered an increase in IL8, MCP1 and/or MIP1β secretion(**Supplementary Figure 10A**, lines with positive impact in green). In opposition, pre-treating tumor cells with soluble UEA-I, RPL-αMan or RPL-Gal2 (blocking Fuc, Fuc/Man or Gal residues respectively) increased the positive impact on cDC1s and pDCs, boosting the production of IFNλ1 by cDC1s and/or IFNα by pDCs (tumor cells #1, #2 and #4 for UEA-I, tumor cells #2 and #4 for RPL- αMan and all tumor cells for RPL-Gal2) when compared to co-culture with untreated tumor cells (**Figure 5B**). Strikingly, blocking Fuc residues on tumor cells (using soluble UEA-I) incredibly enhanced production of cytokines by DCs compared to co-culture with untreated tumor cells (up to 53.7% of IFNλ1^+^ cDC1s compared to 31.53%; up to 26.03% of IFNα^+^ pDCs compared to 11.69%) (**Figure 5C**). Even if limited, this study reveals αGalNAc, GlcNAc and NeuAc as potential booster glycans for cDC1s and Man/Glc for pDCs, whereas Fuc and Gal motifs display rather inhibitory impacts on these DC subsets.

### 4.6 Targeting specific glycans on melanoma tumor cells blocks their negative impact on DC subsets and restored DCs’ functionality

**Figure 6:**
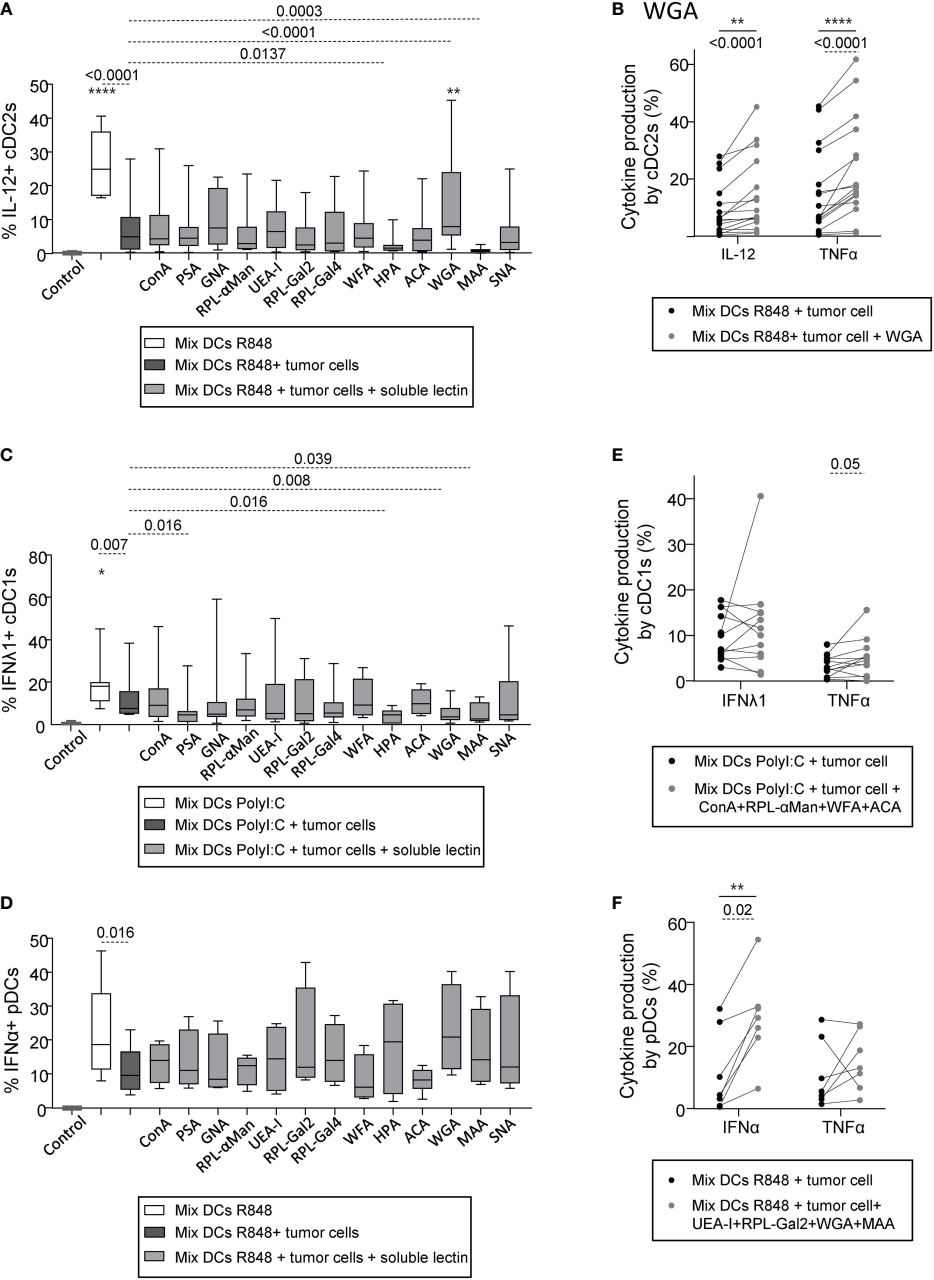
Pre-treatment of “negative” tumor cells with specific lectins *in-vitro* reverses their damaging impact on cDC2s and pDCs’ cytokine production. PanDCs were purified from several HD blood (n=12) and co-cultured for 20 hours with distinct tumor cell lines (n=12) previously cultured or not with single or mixture of soluble lectins for 2 hours. Collected panDCs were then stimulated for 5 hours with or without TLR-L and cytokine production was assessed by intracellular cytokine staining. **(A)** Frequencies of IL-12p40/p70^+^ cDC2s upon R848 after culture (gray and black bars) or not (white bars) with tumor cells previously treated (gray bars) or not (black bars) with single soluble lectins (n = 11 to 20 cocultures per group including 12 tumor lines and 12 HDs). **(B)** Proportions of cytokine-producing (IL-12p40/p70 and TNFα) cDC2s upon R848 stimulation after culture with tumor cell lines treated (gray circles) or not (black circles) with WGA (blocks mostly GlcNAc and NeuAc residues) (n = 16 cocultures per group including 12 tumor lines and 12 HDs). **(C, D)** Proportions of IFNλ1^+^ cDC1s upon PolyI:C stimulation **(C)** (n = 8 to 12 cocultures per group including 8 tumor lines and 7 HDs) and of IFNα^+^ pDCs upon R848 stimulation **(D)** (n = 4 to 7 cocultures per group including 5 tumor lines and 10 HDs) after culture (gray and black bars) or not (white bars) with “negative” tumor cell lines previously treated (gray bars) or not (black bars) with single soluble lectins. **(E)** Proportions of cytokine-producing (IFNλ1 and TNFα) cDC1s upon PolyI:C stimulation after culture with “negative” tumor cell lines treated (gray circles) or not (black circles) with a mixture of lectins containing ConA, RPL-αMan, WFA and ACA (n = 12 cocultures per group including 6 tumor lines and 6 HDs). **(F)** Frequencies of cytokine-producing (IFNα and TNFα) pDCs upon R848 stimulation after culture with “negative” tumor cell lines treated (gray circles) or not (black circles) with a mixture of lectins containing UEA-I, RPL-Gal2, WGA and MAA (n = 7 cocultures per group including 7 tumor lines and 7 HDs). **(A-F)** Results are expressed as percentages of cytokine-expressing cells within each group. Interleaved box and whiskers representation plotting were from minimum to maximum. “Control” represents the condition mix DCs alone (without tumor, without lectin, without TLR-L). Only significant statistics are shown. *P*-values were calculated using both matched two-way repeated measures ANOVA (full lines) or mixed-effects model (REML; stars) with Bonferroni’s multiple comparisons test, and/or Wilcoxon matched-paired signed rank test (dashed lines). Stars represent a significant difference between the given group and the condition “Mix DCs + tumor cells”. *P-value ≤ 0.05, **P-value ≤ 0.01, ****P-value ≤ 0.0001.

As tumor harbored an aberrant glycosylation in melanoma that could potentially be sensed by DCs and impact their functionality, we then explored if the effect of tumor cells with a negative impact on DC subsets could be reversed by blocking tumor glycans. With the goal to identify glycans negatively influencing DCs’ function, we used tumor cell lines displaying a negative impact on DCs, and assessed which lectin would restore DCs’ function. To avoid contact of DCs with lectins, tumor cells were washed to remove any potential remaining lectins. We verified that the “negative” tumor cells pre-treated with soluble lectins had no impact on cytokine production by cDC2s, cDC1s and pDCs in absence of TLR stimulation (condition “w/o stim”) (**Supplementary Figures S11A–C**). Strikingly, for cDC2s stimulated with R848, the initial abrogation of their cytokine production (IL-12p40/p70 and TNFα) by tumor cells was abolished upon pre-treatment with soluble WGA (blocking GlcNAc residues) (**Figures 6A–B**, **S12A**), suggesting that GlcNAc is a potential deleterious glycan responsible for the negative impact of tumor cells on cDC2s upon TLR triggering. Yet, blocking specific glycans such as αGalNAc and NeuAc residues (using soluble HPA or MAA respectively) on tumor cells enhanced the subversion caused by “negative” tumor cells and further decreased cytokine production by cDC2s (**Figure 6A**, **S12A**). Such effects on cDC2s could be also observed in the presence of PolyI:C, as in these settings, soluble WGA restored TNFα production by cDC2s and blocking αGal, αGalNAc or NeuAc residues (using soluble RPL-Gal2, HPA or MAA respectively) decreased the proportions of TNFα^+^ cDC2s (**Supplementary Figure S12B**). Regarding cDC1s and pDCs, the inhibition of cytokine production upon TLR stimulation initially observed after co-culture with “negative” tumor cells could not be abolished by blocking individually glycan residues (**Figures 6C–D**, **S12C–D**), suggesting the existence of multiple deleterious glycans on tumor cells detrimental for cDC1s and pDCs. Interestingly, blocking Man/Glc, αGalNAc, GlcNAc or NeuAc residues (using soluble PSA, HPA, WGA or MAA respectively) on “negative” tumor cells before co-culture with “mix DCs” further decreased frequencies of IFNλ1^+^ cDC1s upon PolyI:C stimulation (**Figure 6C**), further confirming the positive impact of Man/Glc, αGalNAc, GlcNAc and NeuAc motifs on cDC1s previously suspected. Then, to identify deleterious glycans, we simultaneously blocked glycans displaying a tendency to restore DCs’ function upon individual blocking using mixtures of lectin. Pre-treating “negative” tumors with a mix of ConA, RPL-αMan, WFA and ACA (blocking Man/Glc, Fuc/Man, αGalNAc and TF-antigen respectively) before co-culture with “mix DCs” allowed to slightly increase TNFα production by cDC1s after PolyI:C stimulation (**Figure 6E**). Strikingly, pre-treating “negative” tumors with a mix of UEA-I, RPL-Gal2, WGA and MAA (blocking Fuc, Gal, GlcNAc and NeuAc residues respectively) before co-culture with “mix DCs” allowed to revert their negative impact on pDCs and significantly increase proportions of IFNα^+^ pDCs after R848 stimulation (**Figure 6F**). Interestingly, treatment of the “negative” tumor cells with WGA or MAA triggered an increase in IL1β, IL6, IL8 and/or MIP1β secretion(**Supplementary Figure 10A**, lines with negative impact in red). To further decipher if the reversion of DCs’ dysfunction using lectins could be mediated by tumor-derived conditioned medium, we assessed DCs’ cytokine production upon culture with supernatants derived from tumor cell lines pre-incubated with lectins (WGA, HPA, MAA) for cDC2s and pDCs whose functionality was affected by tumor-derived supernatants. For cDC2s, the negative impact of tumor-derived supernatants was not reversed by pre-treatment of tumor cells with the lectins, whereas for pDCs, the negative impact of tumor-derived supernatants may be reversed by pre-treatment of tumor cells with HPA and WGA (**Supplementary Figure 10B**). Thus, targeting specific glycans on tumor cells allows restoring potent DCs’ functionality. In addition to preventing glycan binding on lectins expressed by DCs, reversion of DCs’ dysfunction upon treatment of tumor cells by specific lectins may rely on modification of the secretome of tumor cells.

### 4.7 The tumor glyco-code may dictate the nature and magnitude of the immune infiltrate found in melanoma patients

**Figure 7:**
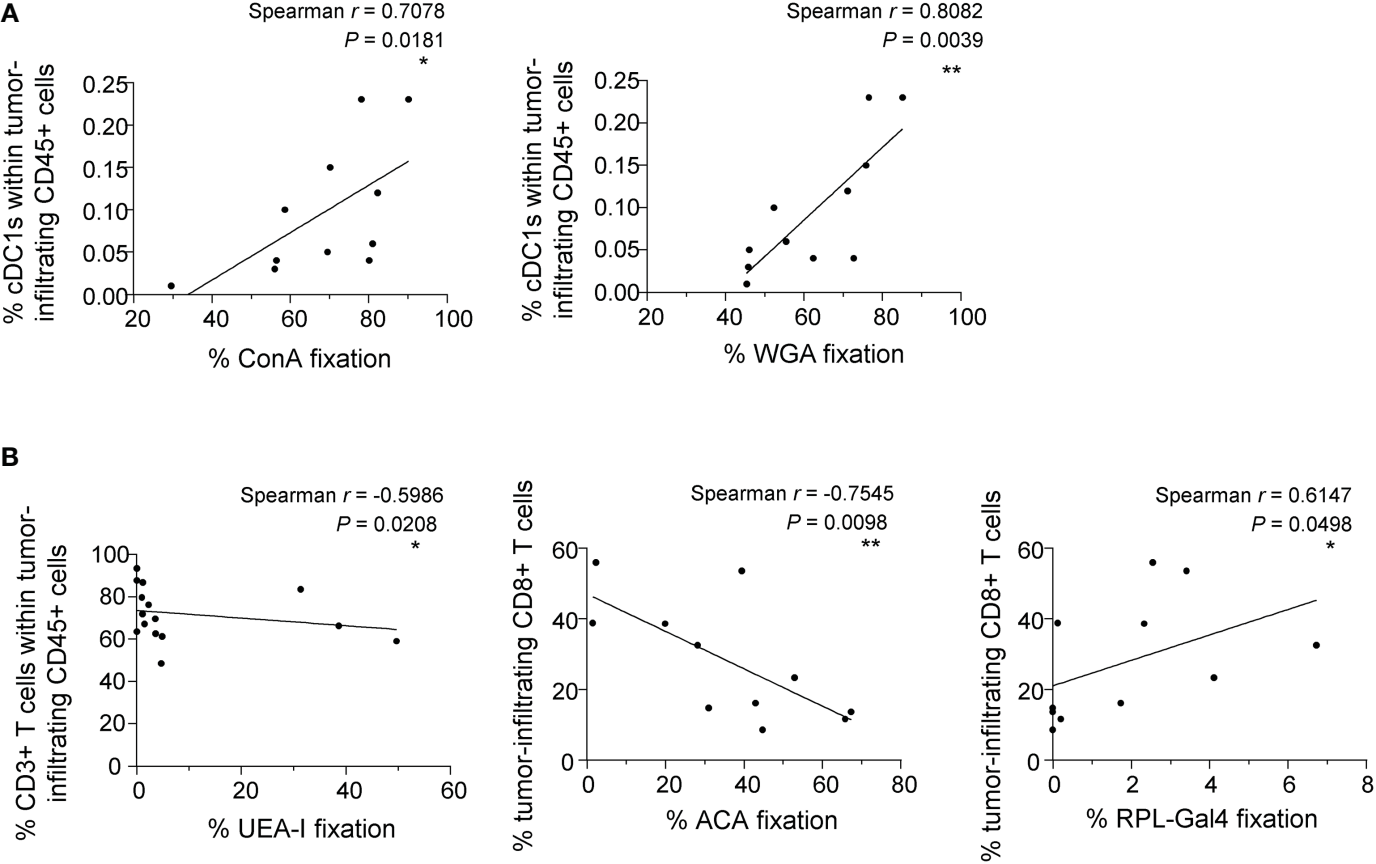
The tumor glyco-code correlates with the nature and magnitude of the immune infiltrate. Proportions of tumor-infiltrating CD45, cDC1s, CD3 and CD8 T cells were evaluated by flow cytometry on the same patients’ tumor samples from whom the glyco-code of derived primary tumor cell lines was performed. Spearman correlation were performed to assess the link between the tumor glyco-code and immune cell infiltration. **(A)** Spearman’s correlation of frequencies of ConA (left panel) or WGA (right panel) interaction with tumor cells lines derived from melanoma patients and proportions of tumor-infiltrating cDC1s within CD45^+^ cells (n=11). **(B)** Spearman’s correlation of frequencies of UEA-I (left panel), ACA (middle panel) or RPL-Gal4 (right panel) binding by tumor cell lines derived from melanoma patients and proportions of tumor-infiltrating CD3^+^ or CD8^+^ T cells (n = 11 to 15). *P-value ≤ 0.05, **P-value ≤ 0.01.

As we previously highlighted the importance of the tumor glyco-code for immune responses in melanoma and its link with patient’s clinical outcome, we further explored whether the tumor glyco-code could be linked with the proportions of tumor-infiltrating immune cells (CD45 cells, DC subsets, T cells) in melanoma patients. We performed Spearman correlations between lectin fixations by tumor cell lines derived from patients (GLYcoPROFILE™ study) and the nature and magnitude of immune infiltrates analyzed on the corresponding tumor samples by flow cytometry (**Supplementary Figure 13**, **Supplementary table 4**). Notably, we found positive correlations between proportions of tumor-infiltrating cDC1s and levels of Man/Glc and GlcNAc residues (seen by ConA and WGA fixation) on tumor cells (**Figure 7A**). Moreover, levels of Fuc residues (seen by UEA-I fixation) on tumor cells negatively correlated with infiltration by T cells (**Figure 7B**; left panel). Interestingly, infiltration of tumors by CD8^+^ T cells negatively correlated with levels of TF-antigen residues (studied by ACA fixation) on tumor cells, while positively correlated with levels of β-Gal residues (seen by RPL-Gal4 fixation) on tumor cell lines from melanoma patients (**Figure 7B**; middle and right panels). Thus, the tumor glyco-code correlates with the nature and abundance of immune infiltrate in melanoma patients, suggesting that aberrant glycosylation of tumors may shape the tumor immune microenvironment using multiple ways.

## 5 Discussion

**Figure 8:**
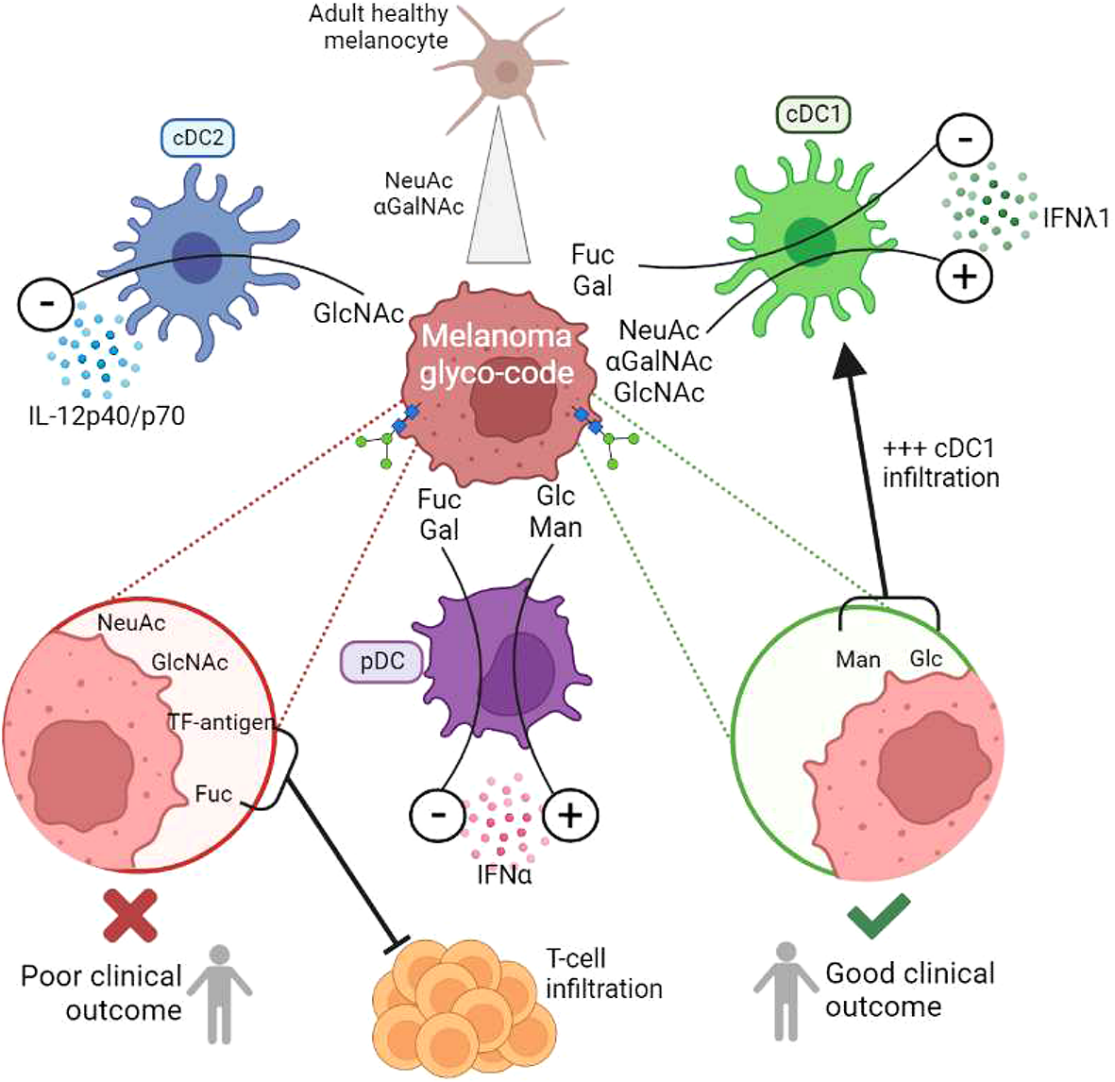
(Graphical summary): Melanoma tumor glyco-code impacts DC subsets’ functionality, dictates the nature of immune infiltrate, and drives patients’ clinical outcome. To decrypt potential links between aberrant glycosylation patterns and immune evasion in melanoma, we explored the melanoma tumor glyco-code through the GLYcoPROFILE™ methodology (lectin arrays), and depicted its impact on patients’ clinical outcome and DC subsets’ functionality. Primary tumor cells derived from melanoma patients harbored a specific glyco-code characterized by high levels of GalNAc and NeuAc residues compared to healthy melanocytes, with heterogeneous expression of Man, Fuc and Gal motifs. Specific glycan patterns on tumor cells correlated with clinical outcome of melanoma patients, especially GlcNAc, NeuAc, TF-Ag and Fuc motifs being associated with poor outcome, whereas Man and Glc residues eliciting better survival. Strikingly, tumor cells which differentially impacted cytokine production by DC subsets harbored distinct glyco-profiles. GlcNAc exhibited a negative influence on cDC2s, whereas Fuc and Gal motifs displayed inhibitory impacts on cDC1s and pDCs. We further identified αGalNAc, GlcNAc and NeuAc as potential booster glycans for cDC1s, and Man/Glc for pDCs. Targeting specific glycans on melanoma tumor cells blocked their negative impact on DC subsets and restored potent DCs’ functionality. The tumor glyco-code was also linked to the nature and magnitude of the immune infiltrate, as levels of Man/Glc and GlcNAc residues positively correlated with proportions of tumor-infiltrating cDC1s, and levels of Fuc and TF-Ag residues negatively correlated with infiltration by T cells.

For the first time, we depicted the global melanoma glyco-code and its impact on immunity, and unravelled that glycans may govern DCs’ functionality and dictate clinical outcomes of patients (**Figure 8**, Graphical abstract). Our study reveals the glycan/CLR axis as a new immune subversion pathway in melanoma, and further paves the way to exploit glycan-lectin interactions for the design of innovative therapeutic strategies.

Using an innovative approach of GLYcoPROFILE™, we first highlighted abnormal glycan patterns on melanoma tumor cells compared to healthy melanocytes. High levels of αGalNAc and NeuAc motifs (revealed by WFA and MAA fixation) were the major changes occurring in melanoma tumor cells compared to healthy melanocytes. In addition, we observed within tumor cell lines a high heterogeneity regarding fucose residues (unveiled by RPL-αMan and UEA-I fixation). This is in line with few studies already reporting changes in the glycome between healthy skin and skin cancers, using different glycomics approaches. The human skin glycome is dominated by simple O-glycans and complex N-glycans exhibiting similar levels of α2,3- and α2,6 sialylation, with exclusive core fucosylation (38). Normal melanocytes displayed abundant I-branched glycans that progressively diminished in primary and metastatic melanoma (22). The most frequent glycosylation changes described in melanoma are sialylation, fucosylation and N- and I-branching glycans (23), demonstrating that melanoma cells display an altered glyco-code.

Our study further highlighted links between the glyco-code and clinical outcomes of melanoma patients. Indeed, within metastatic tumor cell lines, higher levels of GlcNAc, TF-antigen, NeuAc and Fuc motifs were associated with poor outcome, whereas higher levels of Man and Glc were linked with a longer PFS. This is in line with a previous study revealing that an increased α2,3-sialylation was associated with a more aggressive phenotype in melanoma (39). As glycosylation patterns on cell surface proteins and lipids result from an array of glycosyltransferases/glycosidases activities, recent studies focused on studying the potential dysregulation of such enzymes in driving melanoma progression. Interestingly, several specific glycosyltransferases are dysregulated in melanoma: abnormal expression of N-glycan branching enzymes, loss of N-acetylglucosaminyltransferases (GCNT2) (22), increase of sialyl-transferases (ST3/6Gal1), α1,2- (FUT1, FUT2) and α1-6 (FUT8) fucosyltransferases (21), all concurring to promote a pro-metastatic phenotype of tumor cells (19, 23, 24). Such alterations completely remodeled glycan patterns on the surface of melanoma tumor cells, creating incomplete O-glycan structures, increased N-branched and sialylated glycans, and altered fucosylation. Notably, among many lectins differing in their carbohydrate specificity, a study performed on 100 cases of cutaneous malignant melanoma revealed a positive correlation between HPA binding and metastasis, indicating that N-acetyl-galactosamine/glucosamine (GalNAc, GlcNAc) residues are independent predictors for metastasis formation in melanoma (40). In our cohort of metastatic tumor cell lines, HPA binding had a tendency to drive longer progression-free survival, demonstrating that GalNAc and/or GlcNAc motifs (41), despite driving metastasis from primary tumors, may be good predictors in advanced disease.

Although the role of glycans in driving melanoma metastasis has been largely deciphered, the impact of altered glycosylation patterns on immunity has not been explored in melanoma. Yet immune cells express many glycan-binding receptors (lectins) able to sense changes in glycan signatures, such as C-type lectin receptors (CLRs), sialic acid-binding immunoglobulin-like lectins (Siglecs), galectines and selectins. In melanoma, one study described the impact of sialoligands on T cells. Indeed, Siglec-9 engagement by ligands present on melanoma tumor cells suppressed the effector functions of tumor-infiltrating CD8 T cells (42). Our study revealed for the first time that glycan patterns on melanoma tumor cells modulate DCs’ functionality. Strikingly, we demonstrated that tumor cells harboring different glyco-codes differently impacted DCs’ functionality, and that DCs’ dysfunction could be reversed by blocking specific glycans on tumor cells. Interestingly, whereas all tumor cell lines inhibited cDC2s’ function, cDC1s and pDCs were differently sensitive to tumor cells depending on their glyco-code. Such observation may reflect different equipment of DC subsets in lectins. We previously highlighted that DC subsets differently impacted clinical outcomes of melanoma patients with cDC2s displaying altered functionality, pDCs eliciting Th2 and Treg responses, both driving poor survival, while cDC1s preserved potent competences and were associated with improved prognosis (11, 12). The current study further revealed that glycan patterns on tumor cells may influence patients’ clinical outcome through modulation of DCs’ function and subsequent adaptive T-cell responses. Fuc residues negatively modulated cDC1s’ function and were associated with poor clinical outcome, while αGalNAc motifs boosted cDC1s and were linked with a good clinical outcome. Gal residues, by negatively impacting pDCs, may prevent the pDC-triggered Th2/Treg response and further promote CD8 T-cell infiltration.

Further investigating the underlying mechanism of the glycan-mediated effect on DCs would be very relevant. In our previous work (32), we documented a link between glycan motifs on tumor cells and perturbed CLR profiles on tumor-infiltrating DC subsets. The glycoprofile of primary melanoma tumor cell lines revealed high level of Man, Fuc and GlcNAc motifs. These motifs are known to be recognized by Dectin 1 (specific for β-glucans), DCIR, DC-SIGN and CD206 (specific for Man, Fuc and GlcNAc motifs). Strikingly, these CLRs are the one that were the most modulated on tumor-infiltrating DC subsets from melanoma patients (32). We highlighted strong link between specific glycans and corresponding CLR on DCs within the tumor of melanoma patients. Indeed, frequency of tumor-infiltrating DCIR cDC2s positively correlated with level of GlcNAc motifs on corresponding tumor cells. Tumor-infiltrating Dectin1+ cDC1s were linked with Glc motifs on tumor cells. Frequency of tumor-infiltrating DC-SIGN+ cDC2s was negatively linked with lectin recognizing Man motifs. These observations strongly support that specific glycan patterns on melanoma tumor cells may influence DCs’ functionality through CLR binding. Further investigating the role of CLRs but also Siglecs, galectines and selectins in the glycan-mediated effect on DCs would be of major interest.

In other tumor types, there is growing evidence for a role of tumor glycosylation and sialylation in dismantling antitumor defense (26). Aberrant glycan motifs on tumor cells have been described to modulate either negatively or positively immune cells through specific CLRs. Indeed, hyper-sialylated MUC1 on breast tumor cells drove myeloid cells towards immunosuppressive TAMs through engagement of SIGLEC9, leading to tolerance (43). In colorectal carcinoma, Lewis glycans on carcinoembryonic antigen (CEA) impaired moDCs’ function and differentiation upon engagement of DC-SIGN (44). On the opposite, Dectin-1 and MR on macrophages promoted their antitumor activities through recognition of sialylated tumor cells in ovarian carcinoma (45). Recognition of N-glycans structures on tumor cells by Dectin-1 on DCs and macrophages triggered NK-mediated tumor cell killing (46). Moreover, several studies reported a role for sialic acids in protecting tumor cells from immune destruction (47). Hyper-sialylation of melanoma tumor cells facilitated the infiltration by Treg over effector T cells, whereas silencing the sialic acid transporter promoted antitumor response and tumor control *in vivo* in a mouse B16 melanoma model (48). In addition, hyper-sialylation of melanoma suppresses effector functions of tumor-infiltrating Siglec9+ T cells (42). Thus, interactions between tumor glycan motifs and lectins especially CLRs affect immune cells’ function and subsequently modulate antitumor responses.

Glycans can also have an indirect role on alteration of antitumor immunity. Immune checkpoints especially PDL1 on tumor cells are highly glycosylated with N-linked glycans, which contribute to protein stability and promote interaction with its receptor PD1 on T cells, promoting evasion from T-cell immunity (49). Aberrant cancer-associated glycan patterns owns a key role on immune regulation within the tumor microenvironment.

Strikingly, we also unravelled that glycans may dictate the nature of the immune infiltrate. Indeed, we observed positive correlations between levels of Man/Glc and GlcNAc residues on tumor cells and proportions of tumor-infiltrating cDC1s. Man/Glc were linked with a good clinical outcome, and GlcNAc was a candidate to boost cDC1s’ functionality, which is particularly interesting because a high density of cDC1s predicted better outcome (11). Strikingly, levels of Fuc residues on tumors negatively correlated with infiltration by T cells, and were associated with a poor outcome. In addition, levels of TF-antigen residues on tumor cells from melanoma patients negatively correlated with tumors’ infiltration by CD8+ T cells, and were linked to a shorter survival. Thus we highlighted a link between glycan pattern, immune cell infiltration and clinical outcome. Interestingly, a study reported that hyper-sialylation of melanoma tumor cells altered the Teff/Treg balance by influencing NK influx in a B16 mouse melanoma model (48). Therefore glycosylation patterns on melanoma tumor cells may influence the immune composition of the tumor infiltrate and modulate tumor permissivity to immune attack.

As CLRs harbor a critical role in the shaping and orientation of immune responses, the recognition of tumor glycans by CLRs on DCs could induce anti-tumor responses but also trigger immune evasion. Our work illustrates that glycans on tumor cells can be manipulated to restore and even potentiate DCs’ functionality, thus reshaping antitumor immunity while escaping from tumor-induced subversion. Thus exploitation of the glycan/CLR pathways is a promising way to rescue DCs from tumor-induced subversion in melanoma.

Altogether, there is increasing and promising evidence to exploit glycan on tumor cells or glycan/CLR interactions for diagnosis, biomarkers, and therapeutic innovations. As altered tumor cell surface glycosylation occurs in early stage of tumor development, it has been exploited for diagnostic purposes. For instance, detection of circulating glycoproteins expressing specific glycans serves as cancer biomarkers, such as CA19-9 in pancreatic cancer. Fluorescent lectins have been developed for the diagnosis of cancers expressing the corresponding glycans, such as fluorescent MGL to detect Tn-positive tumors (50). N-linked glycosylation could represent a prognostic marker to predict the metastatic potential of melanoma, as a positive correlation between HPA binding and metastasis formation has been highlighted (40). Hence, the tumor glyco-code becomes a promising novel immune checkpoint harnessable for cancer immunotherapy in multiple ways (17): anti-glycan vaccines aiming at inducing specific anti-glycan immune responses, blocking glycans/lectins interactions, or DC targeting through CLR with glycan-coupled tumor antigens. For instance, glycan-modified apoptotic melanoma-derived extracellular vesicles (ApoEVs) harboring DC-SIGN ligands allowed targeting moDCs and efficiently prime antitumor CD8 T cells (51). An efficient targeting of DCs through DC-SIGN using a trifunctional vaccine composed of mannosides conjugated to gp100 antigen and a TLR7 agonist translated into antigen cross-presentation (52). Abnormal cancer-associated glycans could also be targeted with carbohydrate analogs or glycan-specific CAR T cells. Sialic acid sugars are important modulators of the immunosuppressive tumor microenvironment and limit antitumor immunity. Interestingly, it has been shown in a mouse melanoma model that sialic acids blockade using mimetics (Ac53FNeu5Ac) creates a tumor microenvironment permissive for immunotherapy (47), enhancing NK and CD8 T cells’ function while reducing Treg and MDSCs infiltration. Targeting glycosylated antigens on cancer cells could also be achieved using anti‐glycan based CAR T cells. Indeed, genetically engineered T cells expressing siglec7/9‐based CARs are able to recognize sialoglycans and eliminate tumor cells in a non-MHC restricted way (53).

Aberrant glycosylation owns a critical role in melanoma progression, by affecting cell proliferation, invasion, metastasis, and promoting immune evasion. Identifying specific glycan signatures on melanoma tumor cells together with their impact on immunity is a mandatory prerequisite to further understand glycan/lectins immunosuppressive circuits in tumor microenvironment and elaborate new immunotherapeutic opportunities targeting this axis. Our study brings a step forward to the deep understanding of the impact of glycan patterns on immunity, and an additional rationale for targeting aberrant glycosylation in melanoma, opening ways to exploit them for innovative therapeutic options. Glycans/CLRs are emerging as promising immune checkpoints to exploit in order to reshape potent antitumor immunity and inhibit immunosuppressive circuits triggered by aberrant tumor glycosylation patterns, rescuing DCs from tumor’ hijacking and improving clinical success in cancer patients.

## Data availability statement

The original contributions presented in the study are included in the article/**Supplementary Material**. Further inquiries can be directed to the corresponding author.

## Ethics statement

The studies involving human participants were reviewed and approved by the French Blood Service’s (EFS-AuRA) Institutional Review Board and the ethics committee of Grenoble University Hospital (CHU-Grenoble). The patients/participants provided their written informed consent to participate in this study.

## Author contributions

CA conceived the project and supervised the study. CA, ES designed the experiments and wrote the manuscript. ES, CA, BR, LL, MT, FF performed the experiments and analyzed the data. SM, JC, FdF provided patient material and clinical data, and expertise with clinical interpretation of the data; BR, MT, FF, LL, LC provided research input and contributed to data interpretation; BR, SM, FF, LL, LC contributed to manuscript editing. All authors contributed to the article and approved the submitted version.
