# Supplementary material for: The melanoma tumor glyco-code impacts human dendritic cells’ functionality and dictates clinical outcomes
Source: Front Immunol. 2023 Feb 20;14:1120434. doi: 10.3389/fimmu.2023.1120434 (PMC9986448; doi:10.3389/fimmu.2023.1120434)
Supplement: Supplementary file 1 [file DataSheet_1.pdf]

Supplementary Material

1 Supplementary Figure legends

<u>Supplementary figure 1:</u> **Multi-parametric flow cytometry approach to study the impact of tumor cells on cytokine production by DC subsets (cDC2s, cDC1s, pDCs) upon TLR triggering**

Gating strategy depicting the three DC subsets (cDC2s, cDC1s, pDCs) after purification of PanDCs from PBMC. FSC-A and SSC-A parameters allowed the exclusion of cell debris and, after single cells gating using FSC-A and FSC-H parameters, dead cells were excluded using Live and Dead cell staining. Among $CD45^{+}Lin^{-}$ $HLA\text{-}DR^{+}$ cells, cDC2s were defined as $CD11c^{+}BDCA1^{+}$ cells, cDC1s were depicted as $CD11c^{+}BDCA3^{+}$ cells and pDCs were described as $CD11c^{-}BDCA2^{+}$ cells. Representative flow cytometry plots illustrating panDCs purified from HD.

<u>Supplementary figure 2</u>: **Tumor cell lines derived from melanoma patients differentially affected cytokine production by cDCs and pDCs upon TLR triggering**

PanDCs (mixture of the three DC subsets cDC2s, cDC1s, pDCs) were purified from several HD blood and co-cultured with distinct tumor cell lines (derived from melanoma patients) for 20 hours. Collected panDCs were stimulated for 5 hours with or without TLR-L (polyI:C or R848) and the production of cytokines was assessed by intracellular cytokine staining using flow cytometry. (A) Frequencies of TNFα-expressing cDC2s upon TLR triggering after co-culture with (filled circles) or without (open circles) tumor cell lines derived from melanoma patients (n = 13 tumors for cDC2s). (B) Unsupervised

hierarchical clustering of the cell lines, based on the fold change in cytokine production between conditions with and without tumor cells, based on IFNλ1 and TNFα production for cDC1s, and on IFNα and TNFα production for pDCs. The clustering was used to distinguish tumor cells with positive and negative impacts on DCs. (C) Frequencies of TNFα-expressing cDC1s upon TLR triggering after co-culture with (filled circles) or without (open circles) tumor cell lines derived from melanoma patients (n = 5 to 9 tumors per group for cDC1s). Groups were separated according to the positive or negative impact of tumor cells on IFNλ1 production (Figure 1E). (D) Frequencies of cytokine-expressing cDC2s, cDC1s and pDCs upon TLR triggering after co-culture with (filled circles) or without (open circles) tumor cell lines derived from melanoma patients. For each cell line, two to four donors of PanDCs were assessed. For cDC2s, four negative cell lines are shown. For cDC1s and pDCs, two negative and two positive cell lines are displayed. Results are expressed as percentages of cytokine-expressing cells within the corresponding DC subset. (A, C) Results are expressed as percentages of TNFα-expressing cells within the corresponding DC subset. Only significant statistics are shown on graphs. *P*-values were calculated using matched two-way repeated measures ANOVA with Bonferroni's multiple comparisons test (full lines). ****P-value ≤ 0.0001.

<u>Supplementary figure 3</u>: **Tumor-derived supernatants partially mediate the negative impact of tumor cells on DCs' function**

(A, B) Comparative impact of tumor cells or tumor-derived supernatants on DCs' functionality. PanDCs (mixture of the three DC subsets cDC2s, cDC1s, pDCs) were purified from several HD blood and co-cultured with primary tumor cell lines (derived from melanoma patients) or with the corresponding tumor-derived supernatants for 20 hours. Tumor cell lines were selected based on their "negative" or "positive" impact on IL12, IFNλ1 or IFNα production by cDC2s, cDC1s and pDCs

respectively. Collected panDCs were stimulated for 5 hours with or without TLR-L (polyI:C or R848) and the production of cytokines was assessed by intracellular cytokine staining using flow cytometry. Frequencies of cytokine-expressing cDC2s, cDC1s and pDCs upon TLR triggering after co-culture in control conditions, with positive (A, n=3 cell lines) or negative (B, n=3 cell lines) tumor cell lines or their corresponding tumor-derived supernatants. Results are expressed as percentages of cytokine-expressing cells within the corresponding DC subset. (C) Composition of tumor-derived supernatants assessed by Luminex. Levels of IL1β, IL6, IL8, IL10, MCP1, MIP1α, MIP1β and TGFβ were measured by LUMINEX in the supernatants of tumor lines displaying a negative (red, n=3) or positive (green, n=3) impact on DCs' functionality.

<u>Supplementary figure 4:</u> **Tumor cell lines derived from melanoma patients exhibit differences in their glyco-code depending on their localization of origin (cutaneous or lymph node metastasis)**

Primary tumor cell lines derived from metastases excised from melanoma patients were cultured and GLYcoPROFILE™ (lectin arrays from GLYcoDiag) were performed. Samples were then separated given the tumor's initial localization (cutaneous or lymph node metastasis). (A) Heat map based on frequencies of 16 different lectins fixation (binding different glycans) on tumor cell lines derived from cutaneous (n = 6) or lymph node metastasis (n = 14) excised from melanoma patients. (B) Levels of lectin fixation (indicators of glycan expression levels) by tumor cells derived from cutaneous (open circles; n = 5 to 6) or lymph node metastasis (black circles; n = 12 to 14). Results are expressed as percentages of lectin binding within each group. Bars indicate median. Only significant statistics are shown on graphs. *P*-values were calculated using Mann-Whitney non parametric test (dashed lines).

Supplementary figure 5: Tumor cell lines derived from melanoma patients with different clinical outcomes display differences in their glyco-code

After cell culture, GLYcoPROFILE™ (lectin arrays from GLYcoDiag) were performed on tumor cell lines derived from melanoma patients. Samples were then separated given patient's clinical data. (A) Heat map based on frequencies of 16 different lectins fixation (binding different glycans) on tumor cell lines derived from patients with better (n = 6) or worse (n = 5) progression-free survival (PFS) from sampling time (separation based on the median which is 12 months). (B) Frequencies of lectin fixation (indicators of glycan expression levels) by tumor cells derived from patients with better (n = 4 to 6) or worse (n = 4 to 5) progression-free survival (from sampling time). Results are expressed as percentages of lectin binding within each group. Interleaved box & whiskers representation plotting from minimum to maximum. Only significant statistics are shown on graphs. *P*-values were calculated using Mann-Whitney non parametric test (dashed lines).

Supplementary figure 6: Tumors with a positive impact on both cDC1s and pDCs' functionality upon TLR triggering exhibit no common significant difference in their tumor glyco-code

After cell culture, GLYcoPROFILE™ were performed on tumor cell lines derived from melanoma patients. Samples were then separated depending on their individual impact (positive or negative) on cytokine production by cDC1s and pDCs. (A) Heat map based on the frequencies of fixation of 16 different lectins on tumor cell lines derived from patients. Tumors were separated given their positive or negative impact on cytokine production by cDC1s (left panel) and pDCs (right panel) (n = 5 to 8 tumors per group). (B) Frequencies of lectin fixation by tumor cells which positively or negatively impacted cytokine production by cDC1s and pDCs (n = 4 to 6 per group). Results are expressed as percentages of lectin binding within each group. Interleaved bars representation plotting median with

interquartile range. Only significant statistics are shown on graphs. *P*-values were calculated using Mann-Whitney non parametric test (dashed lines).

<u>Supplementary figure 7</u>: Experimental design to assess the potential of specific glycans in triggering or inhibiting DC subsets' functionality

PanDCs were co-cultured for 20 hours with "positive" or "negative" tumor cell lines previously cultured or not with single or mixture of soluble lectins (blocking specific glycans) for 2 hours. Collected panDCs were then stimulated for 5 hours with or without TLR-L (polyI:C, R848) and cytokines' production was measured using flow cytometry. The comparison of cytokine production with and without lectins allows deciphering the involvement of specific glycans in triggering or inhibiting DCs' functionality.

<u>Supplementary figure 8:</u> Pre-treatment of "positive" tumor cells with soluble lectins had no significant effect on cytokine production by DCs without TLR stimulation

PanDCs were co-cultured for 20 hours with "positive" tumor cell lines previously cultured or not with single soluble lectins for 2 hours. Collected panDCs were then stimulated for 5 hours with or without TLR-L (polyI:C, R848) and cytokines' production was measured using flow cytometry. (A) Frequencies of TNFα^+ cDC1s (left panel) or pDCs (right panel) upon TLR triggering after co-culture with (filled circles) or without (open circles) tumor cell lines that positively impacted cDC1s or pDCs' functionality (called "positive" tumors) and that were previously untreated with soluble lectins (n = 3 to 4 different panDC/tumor combos per group). (B) Proportions of IFN$\lambda1^+$ (top panel) or TNFα^+ (bottom panel) cDC1s after 20 hours of culture with (gray and black bars) or without (white bars) "good" tumor cell lines previously treated (gray bars) or not (black bars) with soluble lectins (n = 3 per

group) in absence of TLR stimulation. (C) Frequencies of IFNα$^+$ (top panel) or TNFα$^+$ (bottom panel) pDCs after 20 hours of culture with (gray and black bars) or without (white bars) "good" tumor cell lines previously treated (gray bars) or not (black bars) with soluble lectins (n = 4 per group) in absence of TLR stimulation. (A-C) Results are expressed as percentages of cytokine-expressing cells within each group. Interleaved box & whiskers representation plotting from minimum to maximum. Only significant statistics are shown on graphs. *P*-values were calculated using matched two-way repeated measures ANOVA (full lines) with Bonferroni's multiple comparisons test, or Wilcoxon matched-paired signed rank test (dashed lines). *P-value ≤ 0.05.

Supplementary figure 9: Pre-treatment of "positive" tumor cells with specific lectins *in-vitro* further boosted their good impact on cytokine production by cDC1s and pDCs

PanDCs were co-cultured for 20 hours with "positive" tumor cell lines previously cultured or not with soluble lectins for 2 hours. Collected panDCs were then stimulated for 5 hours with or without TLR-L (polyI:C, R848) and cytokines' production was measured using flow cytometry. Proportions of TNFα$^+$ cDC1s (left panels) and TNFα$^+$ pDCs (right panels) upon PolyI:C or R848 stimulation respectively after 20h of culture or not with "positive" tumors previously treated or not with soluble lectins (n = 3 or 4 tumors). Lectin fixation by each tumor cell line (#1 to 4) was illustrated on the left part and color scaling was done per lectin.

Supplementary figure 10: Reversion of DCs' dysfunction upon treatment of tumor cells by specific lectins may rely on modification of the secretome of tumor cells

A/ Impact of lectins on tumor cells. Tumor cells (6 in total, 3 with positive (green) impact and 3 with negative (red) impact on both cDC1s and pDCs) were incubated with lectins (WGA, HPA, MAA) for 2h, washed, and further cultured for 20h. Factors known to potentially influence DCs' activation or functionality were then quantified in the supernatants by Luminex (IL1β, IL6, IL8, IL10, MCP1, MIP1α, MIP1β, TGFβ). B/ DCs' cytokine production upon culture with supernatants derived from "negative" tumor cell lines pre-incubated with lectins (WGA, HPA, MAA). PanDCs (mixture of the three DC subsets cDC2s, cDC1s, pDCs) were purified from several HD blood and co-cultured for 20 hours with supernatants derived from tumor lines pre-incubated with lectins (WGA, HPA, MAA). Tumor cell lines were selected based on their "negative" impact on IL12 and IFNα production by Cdc2s and pDCs respectively. Collected panDCs were stimulated for 5 hours with or without TLR-L (polyI:C or R848) and the production of cytokines was assessed by intracellular cytokine staining using flow cytometry. Frequencies of cytokine-expressing cDC2s and pDCs upon TLR triggering after co-culture in control conditions or tumor-derived supernatants. Results are expressed as percentages of cytokine-expressing cells within the corresponding DC subset.

Supplementary figure 11: Pre-treatment of "negative" tumor cells with soluble lectins had no significant effect on cytokine production by DCs without TLR stimulation

PanDCs were co-cultured for 20 hours with distinct tumor cell lines previously cultured or not with single soluble lectins for 2 hours. Collected panDCs were cultured for 5 hours without (w/o stim) TLR-L and cytokines' production was assessed by intracellular cytokine staining using flow cytometry. (A) Frequencies of IL-12p40/p70$^+$ (top panel) or TNFα$^+$ (bottom panel) cDC2s after 20 hours of culture with (gray and black bars) or without (white bars) tumor cells previously treated (gray bars) or not (black bars) for 2 hours with soluble lectins in absence of TLR stimulation (n = 11 to 20 per group). (B) Proportions of IFNλ1$^+$ (top panel) or TNFα$^+$ (bottom panel) cDC1s after 20 hours of culture with

(gray and black bars) or without (white bars) “negative” tumor cells previously treated (gray bars) or not (black bars) with soluble lectins (n = 8 to 12 per group) in absence of TLR stimulation. (C) Frequencies of IFNα$^+$ (top panel) or TNFα$^+$ (bottom panel) pDCs after 20 hours of culture with (gray and black bars) or without (white bars) “negative” tumor cells previously treated (gray bars) or not (black bars) with soluble lectins (n = 4 to 7 per group) in absence of TLR stimulation. (A-C) Results are expressed as percentages of cytokine-expressing cells within each group. Interleaved box & whiskers representation plotting from minimum to maximum. “Only significant statistics are shown on graphs. *P*-values were calculated using mixed-effects model (REML; stars) with Bonferroni’s multiple comparisons test, and/or Wilcoxon matched-paired signed rank test (dashed lines).

<u>Supplementary Figure 12</u>: Pre-treatment of tumor cells with WGA lectin *in-vitro* reverses their negative impact on cDC2s’ TNFα production upon TLR stimulation

PanDCs were co-cultured for 20 hours with distinct tumor cell lines previously cultured or not with single soluble lectins for 2 hours. Collected panDCs were cultured for 5 hours without (control) or with TLR-L (poly:IC or R848) and cytokines’ production was assessed by intracellular cytokine staining using flow cytometry. (A) Proportions of TNFα$^+$ cDC2s upon R848 after culture or not (white bars) with tumor cells previously treated (gray bars) or not (black bars) with soluble lectins (n = 11 to 20 per group). (B) Frequencies of IL-12p40/p70$^+$ (top panel) or TNFα$^+$ (bottom panel) cDC2s upon PolyI:C after culture (gray and black bars) or not (white bars) with tumor cells previously treated (gray bars) or not (black bars) with soluble lectins (n = 11 to 15 per group). (C) Proportions of TNFα$^+$ cDC1s upon PolyI:C after culture (gray and black bars) or not (white bars) with “negative” tumor cells previously treated (gray bars) or not (black bars) with soluble lectins (n = 8 to 12 per group). (D) Frequencies of TNFα$^+$ pDCs upon R848 after culture (gray and black bars) or not (white bars) with “negative” tumor

cells previously treated (gray bars) or not (black bars) with soluble lectins (n = 4 to 7 per group). Results are expressed as percentages of cytokine-expressing cells within each group. Interleaved box & whiskers representation plotting from minimum to maximum. "Control" represent the condition mix DCs without any TLR stimulation. Only significant statistics are shown on graphs. *P*-values were calculated using mixed-effects model (REML; stars) with Bonferroni's multiple comparisons test, and/or Wilcoxon matched-paired signed rank test (dashed lines). Stars represent a significant difference between the given group and the condition "Mix DCs + tumor cells". *P-value $\leq$ 0.05, **P-value $\leq$ 0.01, ***P-value $\leq$ 0.001, ****P-value $\leq$ 0.0001.

Supplementary Figure 13: Gating strategy to depict tumor-infiltrating immune cells by multi-parametric flow cytometry

Gating strategy to analyze cDC1s and CD3+ and CD8+ T cells within tumor-infiltrating immune cells. FSC-A and SSC-A parameters allowed the exclusion of cell debris and, after single cells gating using FSC-A and FSC-H parameters, dead cells were excluded using Live and Dead cell staining. Among total immune $CD45^+$ cells, cDC1s were depicted within Lin^- $HLA\text{-}DR^+$ cells as $CD11c^+BDCA3^+$ cells, and T cells identified as CD45+ CD3+ cells among which we further depicted CD8+ T cells. Representative flow cytometry plots for patient #18.

2 Supplementary Tables

Suppl Figure 1

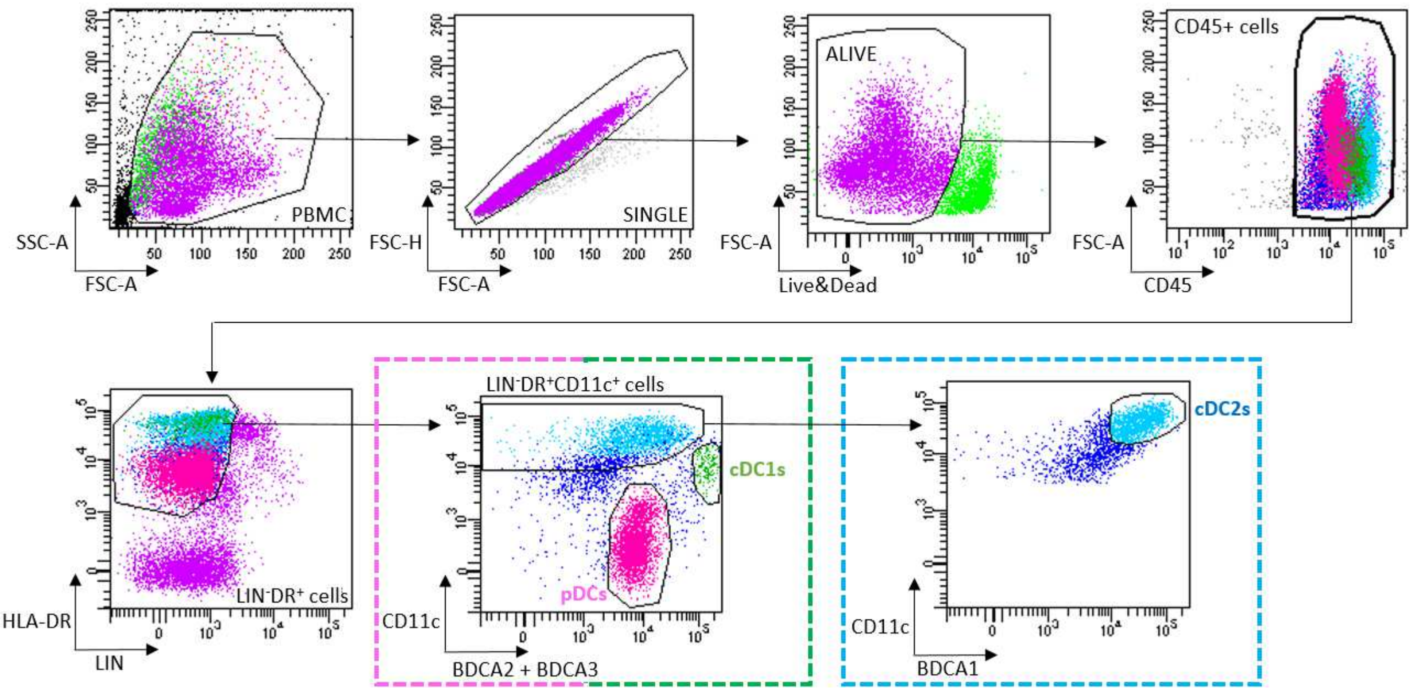

Suppl Figure 2

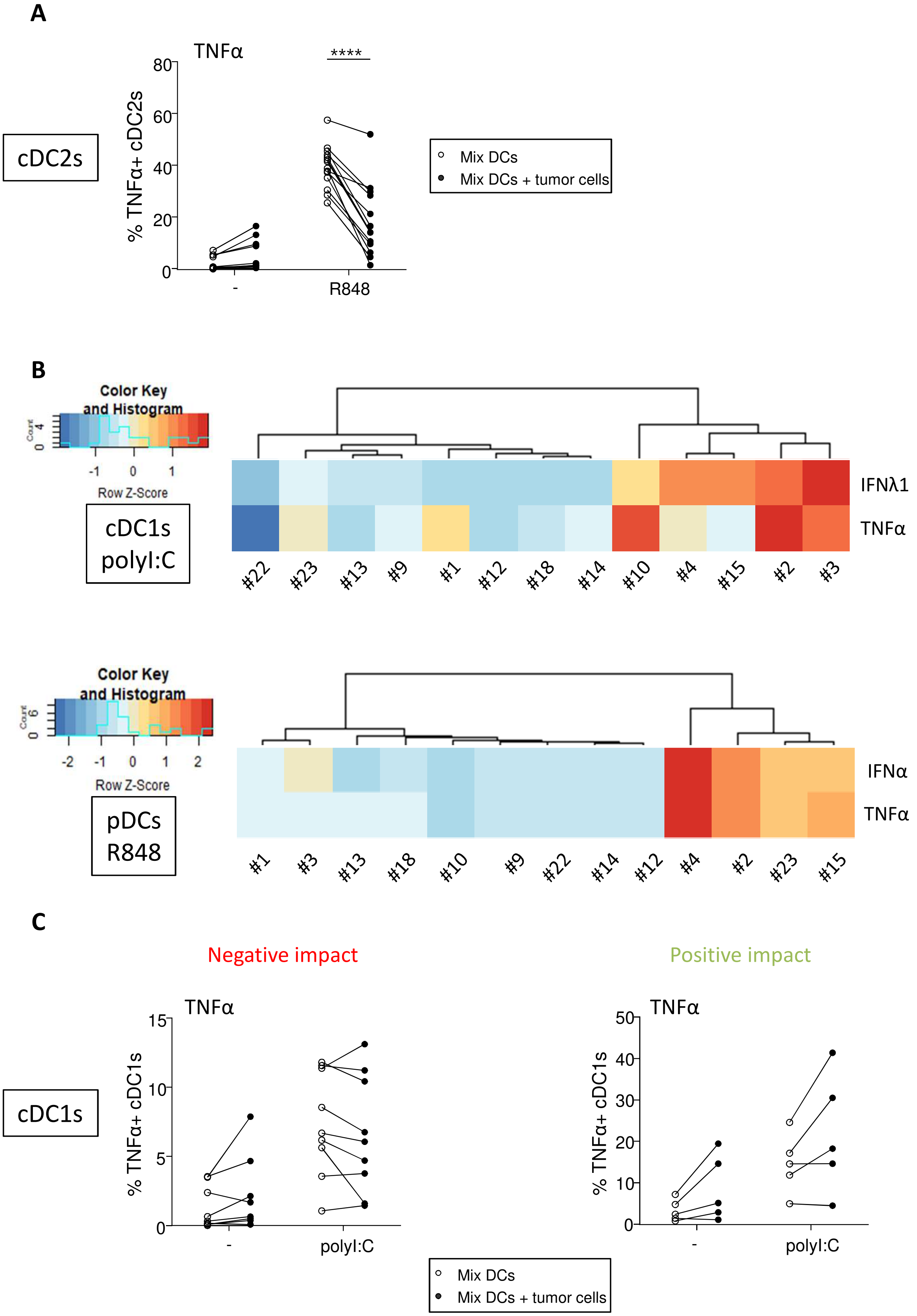

D

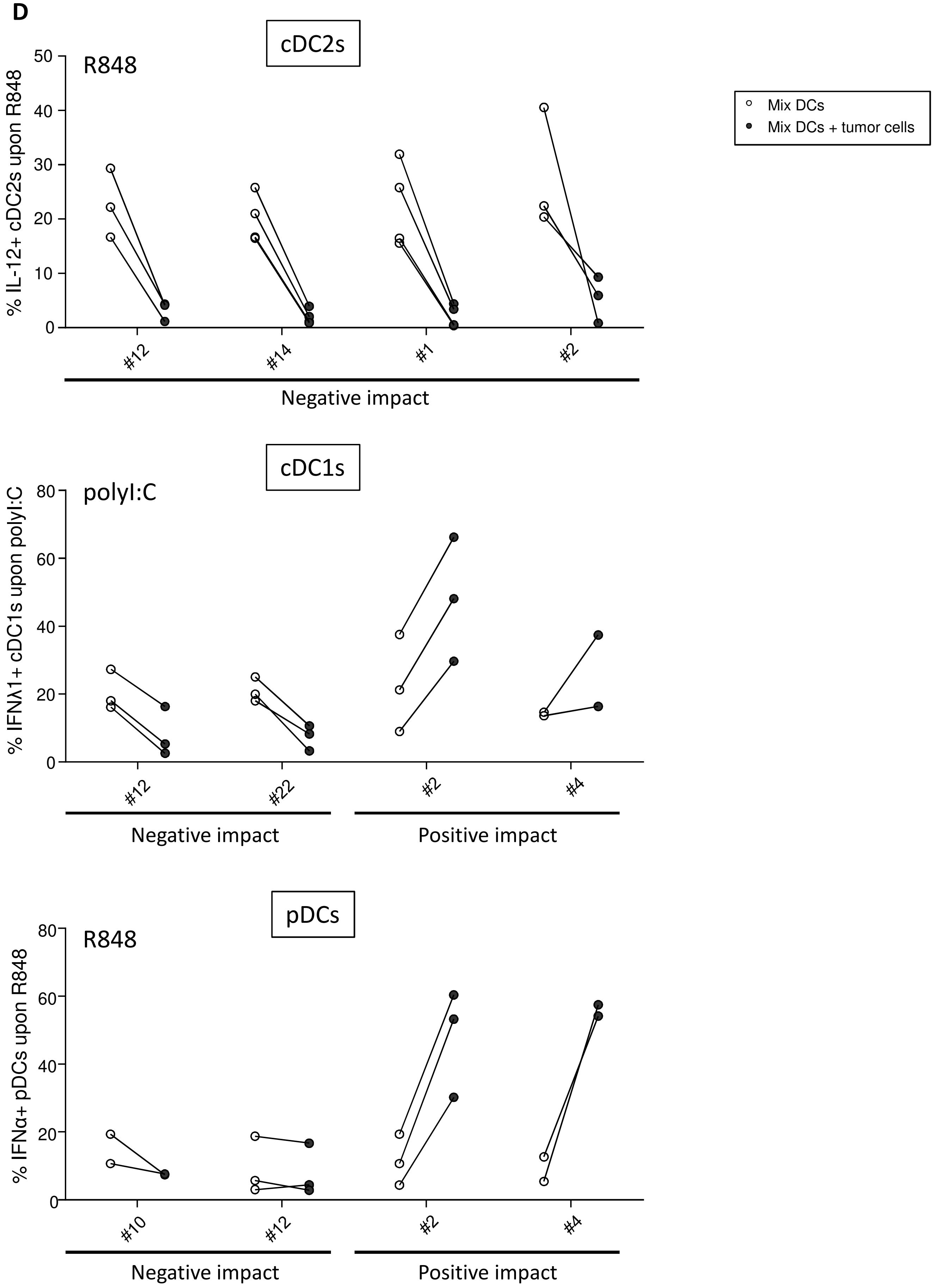
cDC2s
R848
% IL-12+ cDC2s upon R848
50
40
30
20
10
0
#12
#14
#1
#2
Negative impact
Mix DCs
Mix DCs + tumor cells
cDC1s
polyI:C
% IFNλ1+ cDC1s upon polyI:C
80
60
40
20
0
#12
#22
#2
#4
Negative impact
Positive impact
pDCs
R848
% IFNα+ pDCs upon R848
80
60
40
20
0
#10
#12
#2
#4
Negative impact
Positive impact

Suppl Figure 3

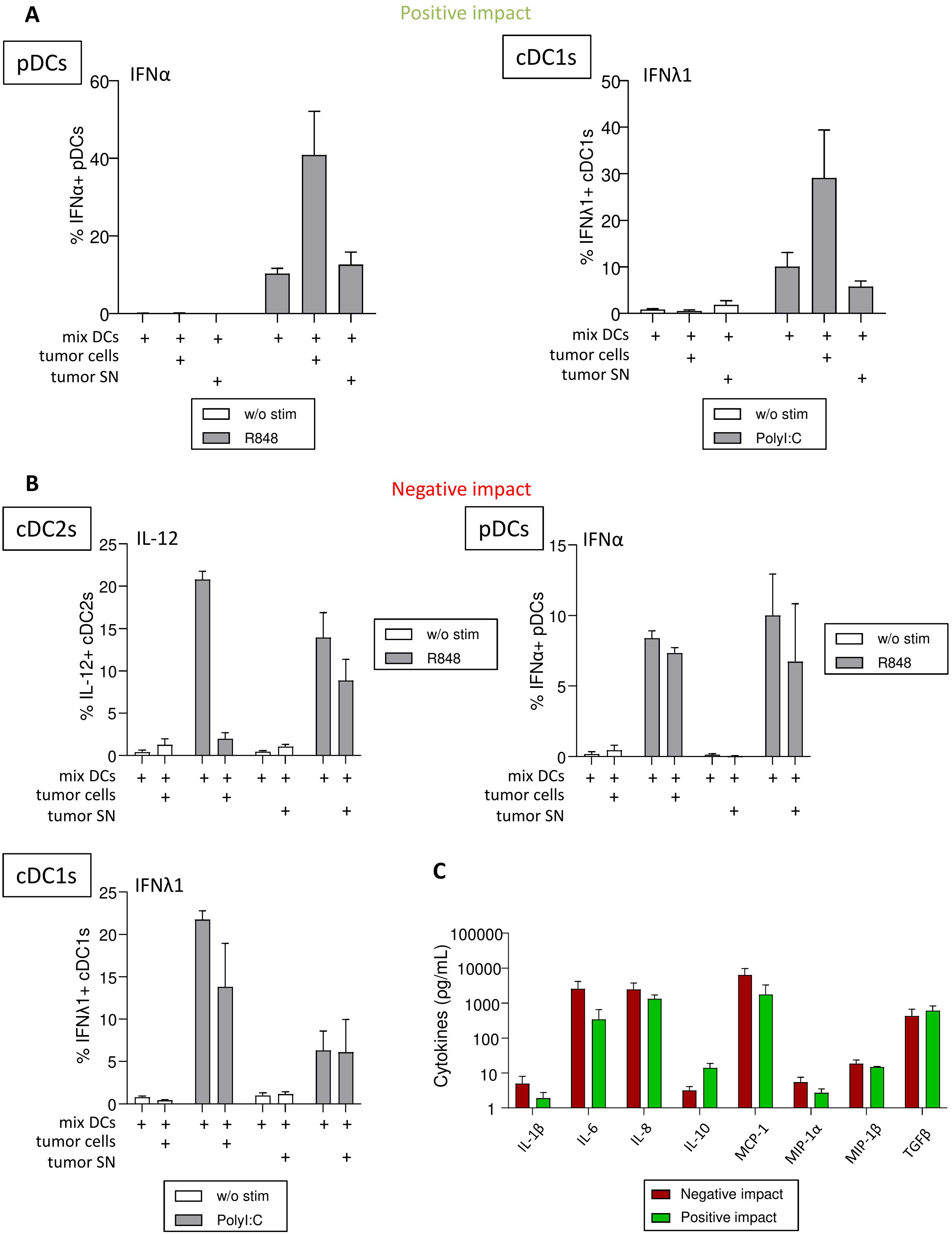

Suppl Figure 4

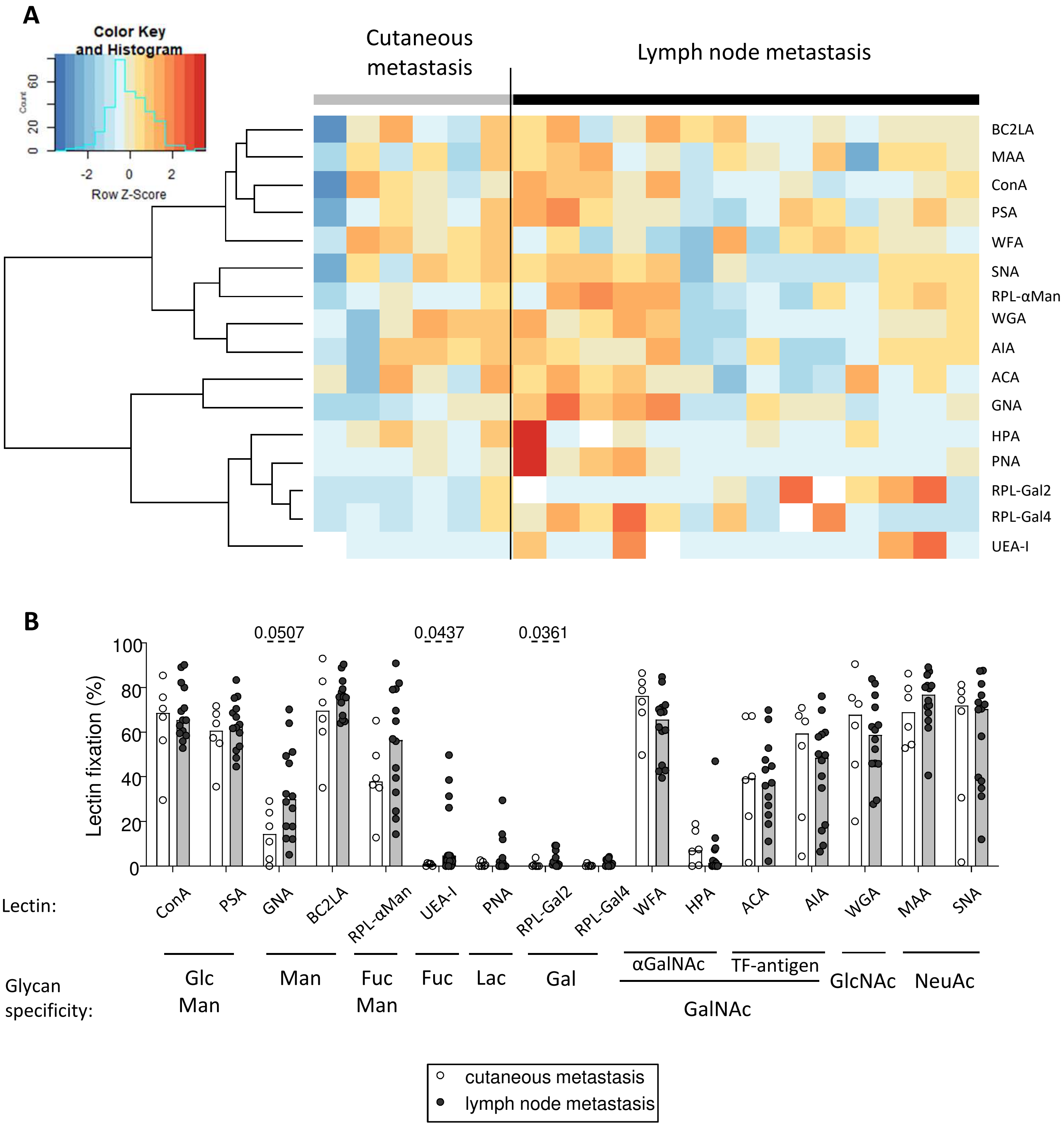

Suppl Figure 5

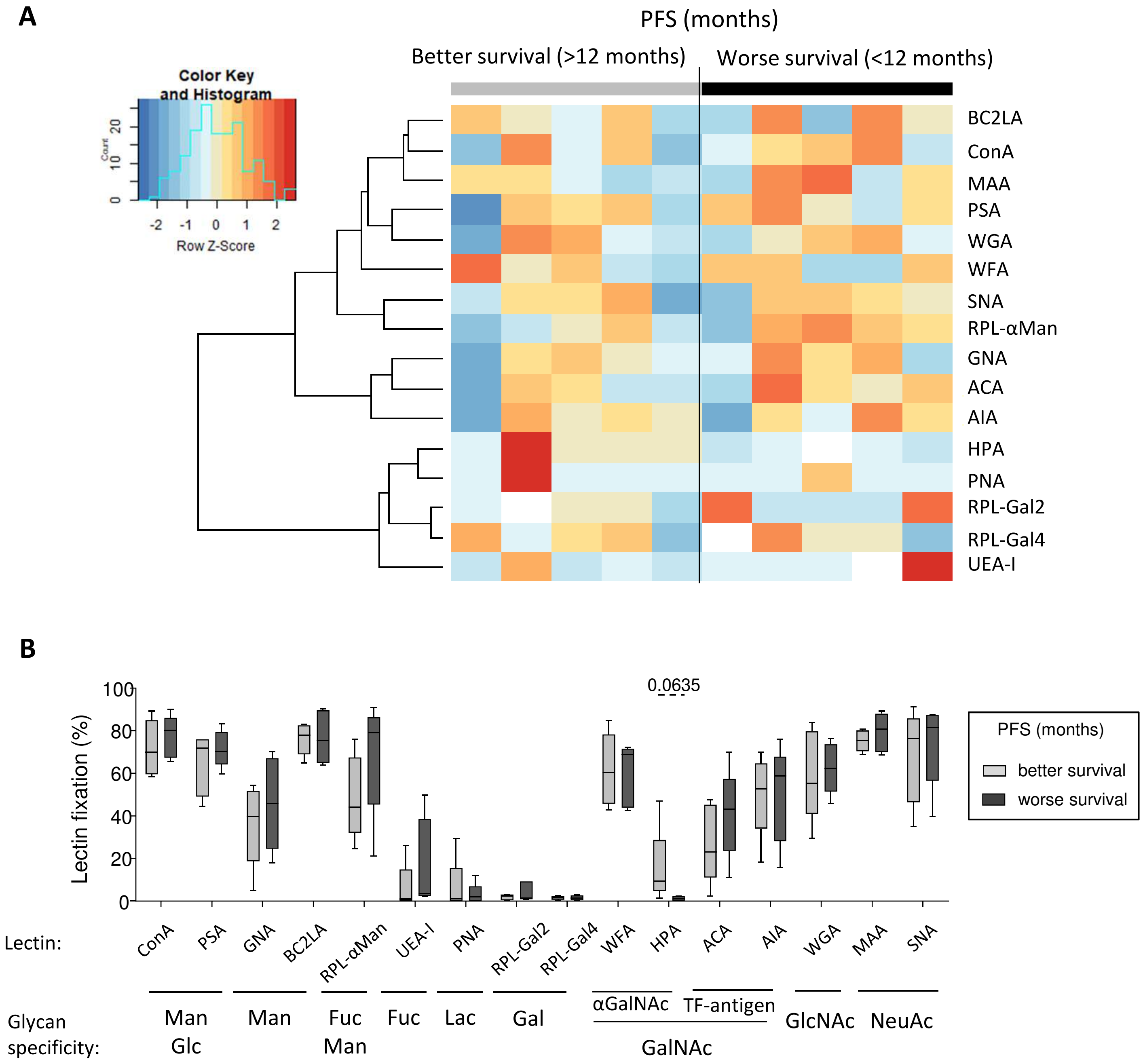

Suppl Figure 6

A

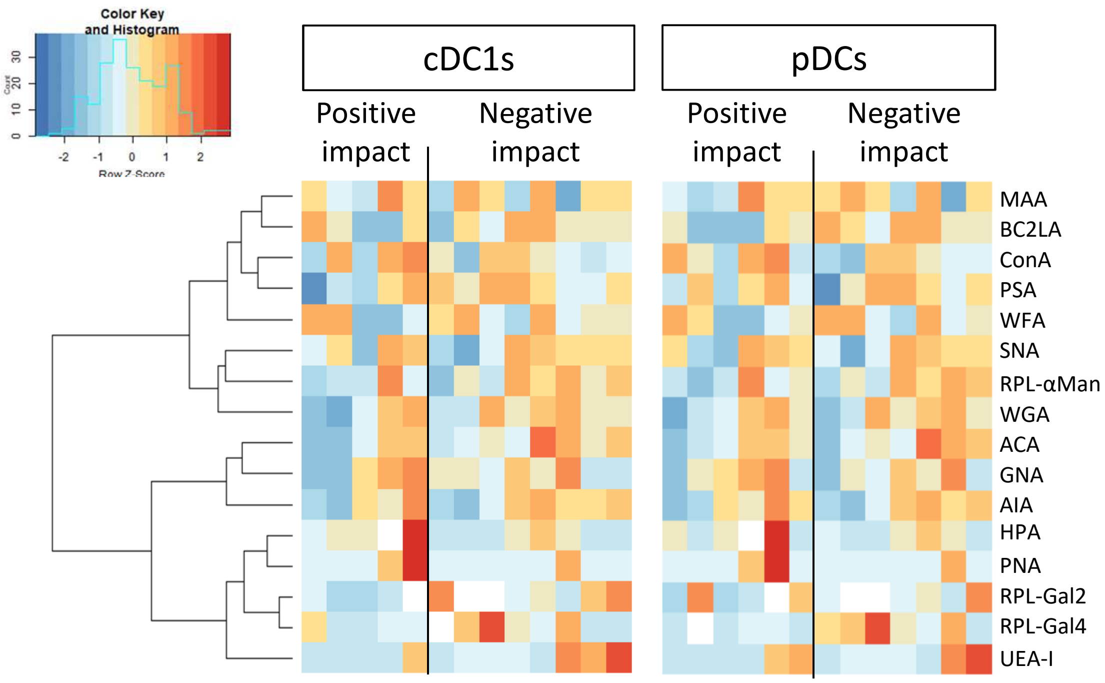

B

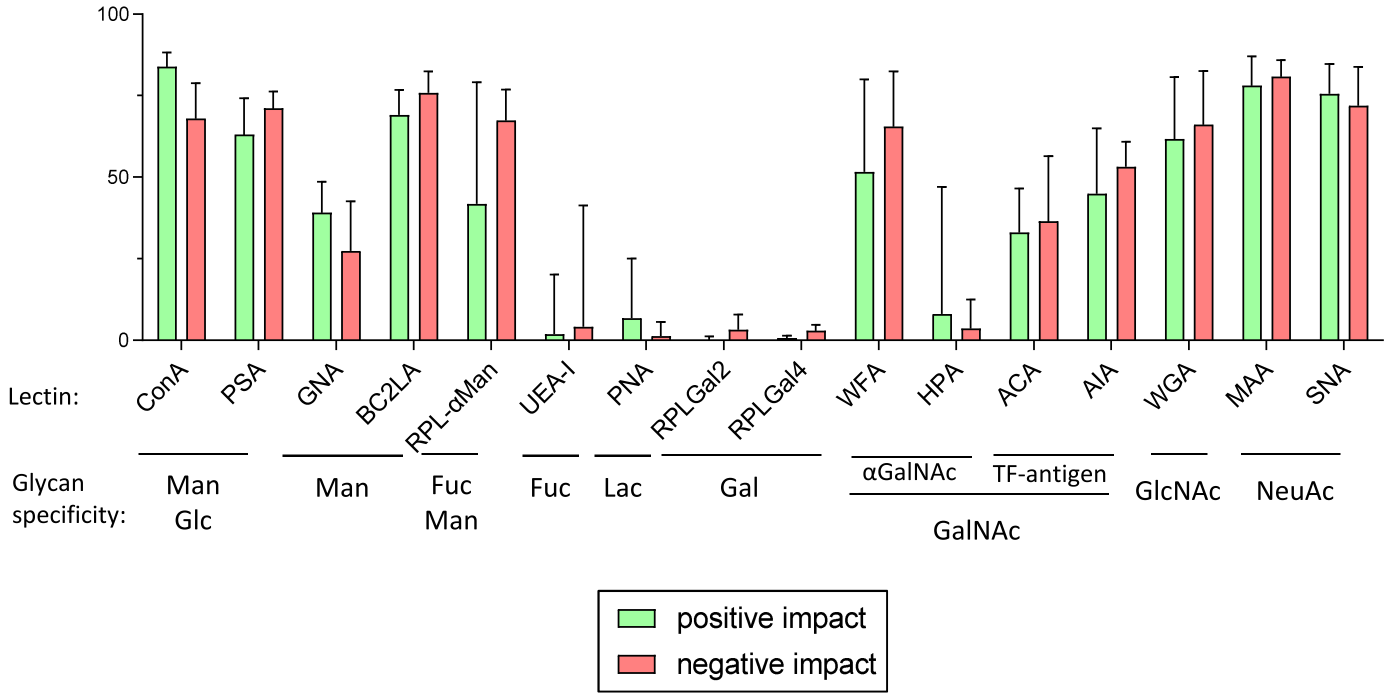

Suppl Figure 7

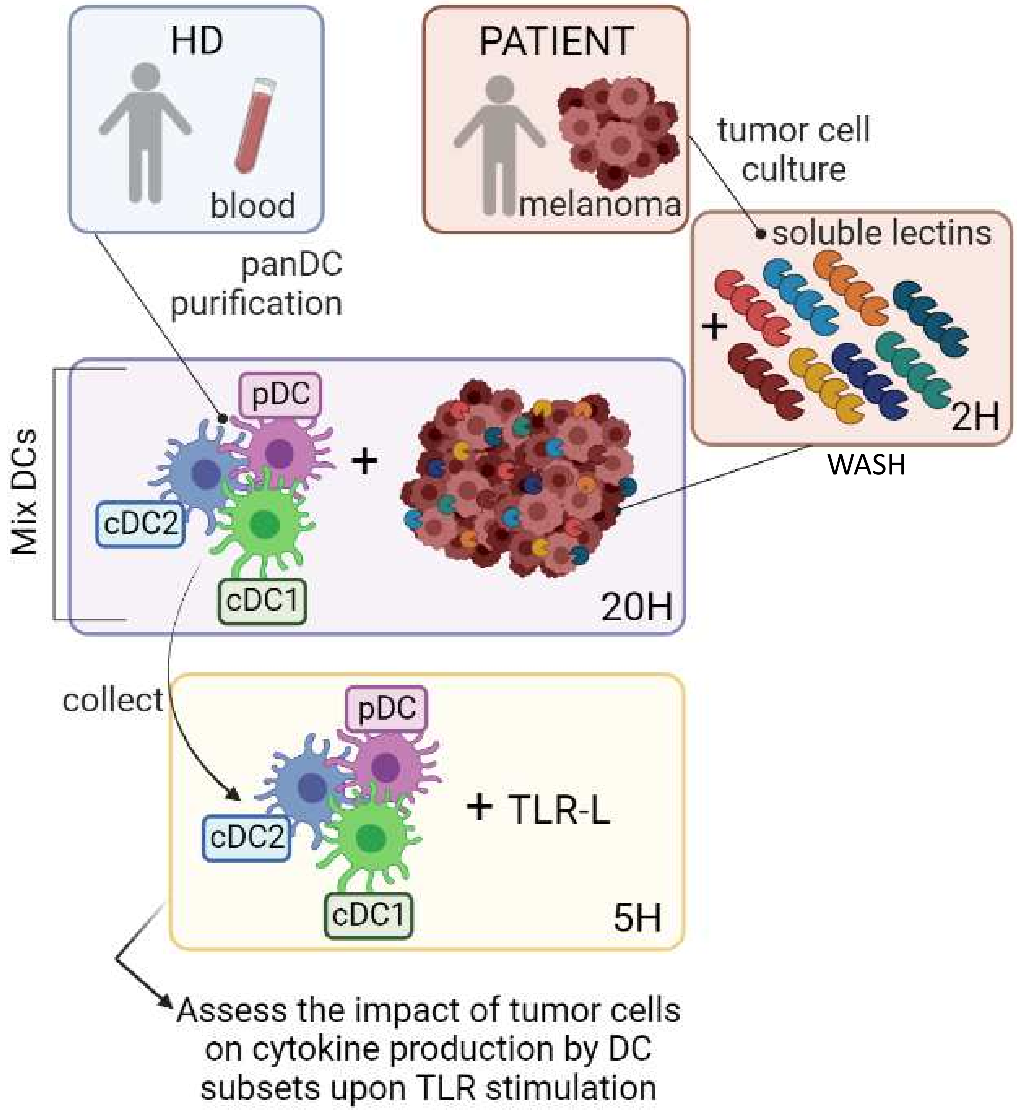

Suppl Figure 8

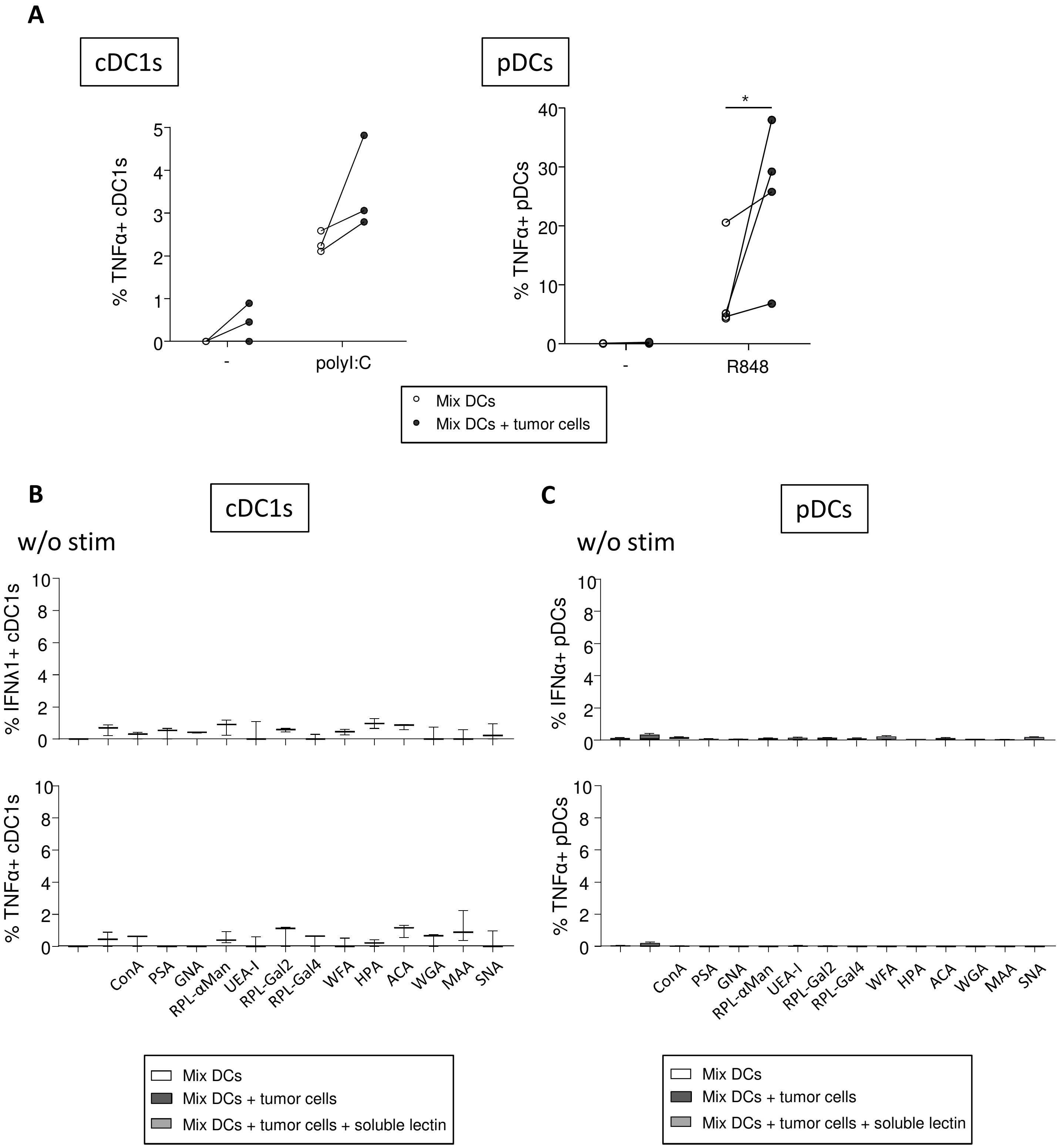

Suppl Figure 9

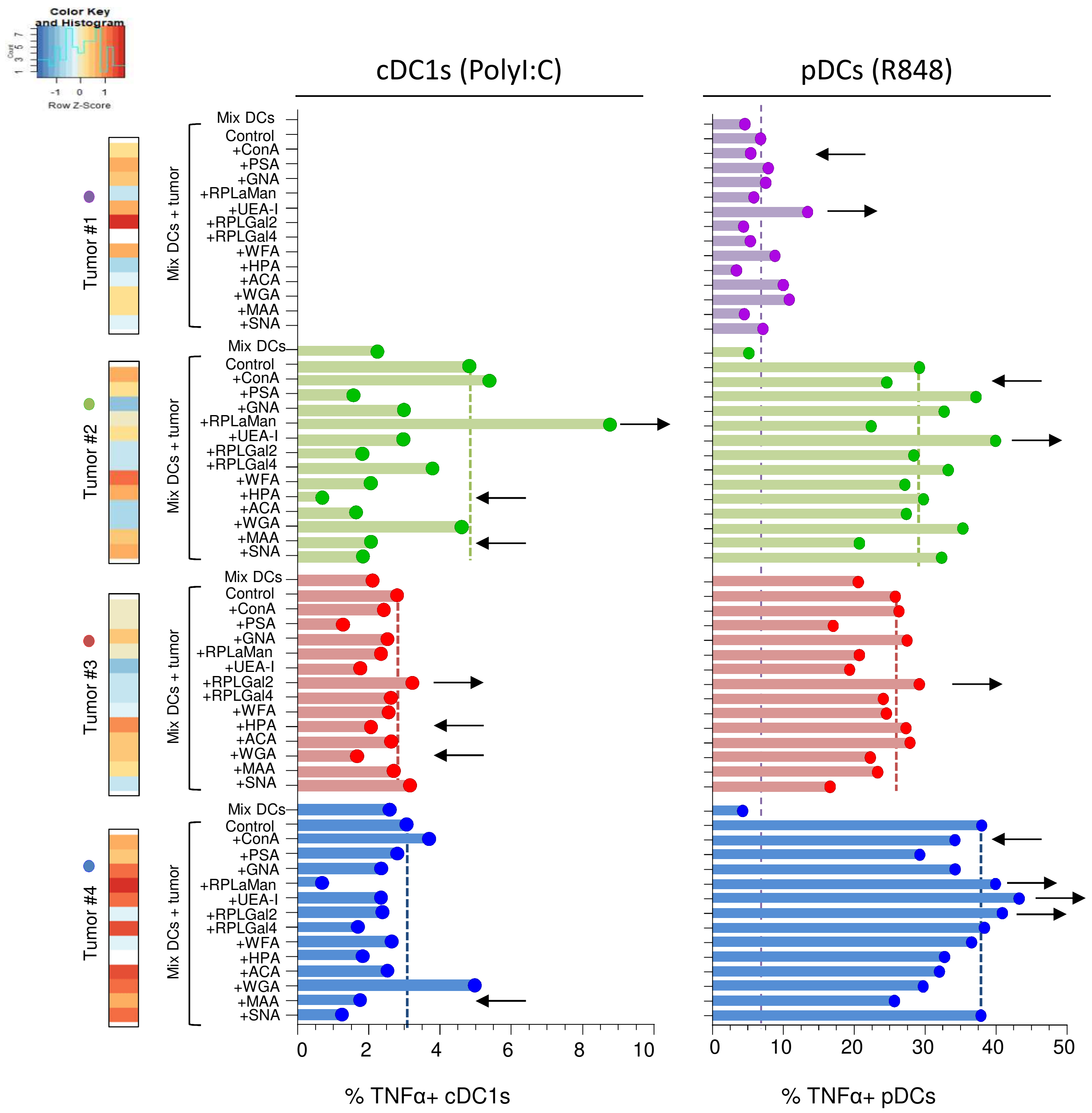

Suppl Figure 10

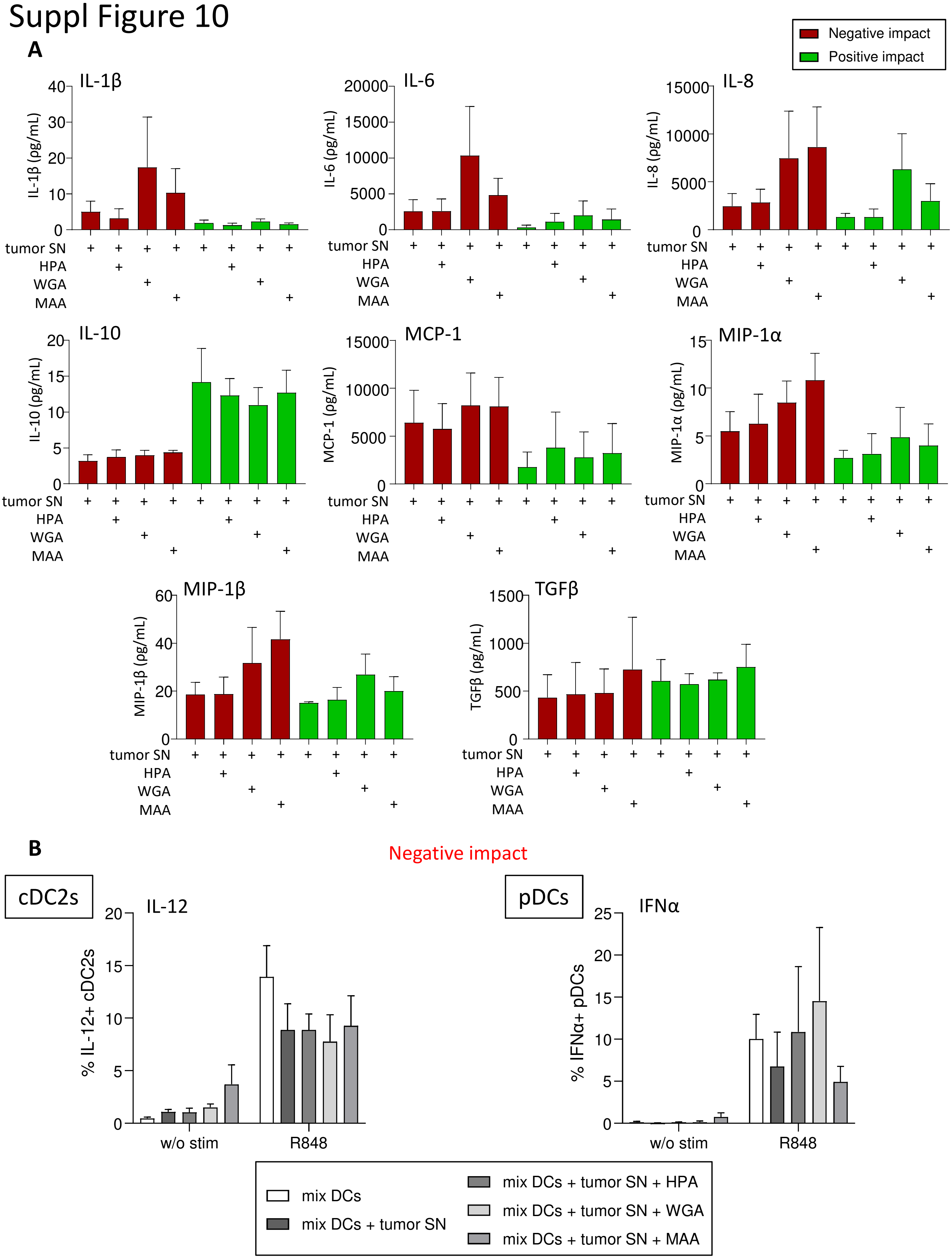

Suppl Figure 11

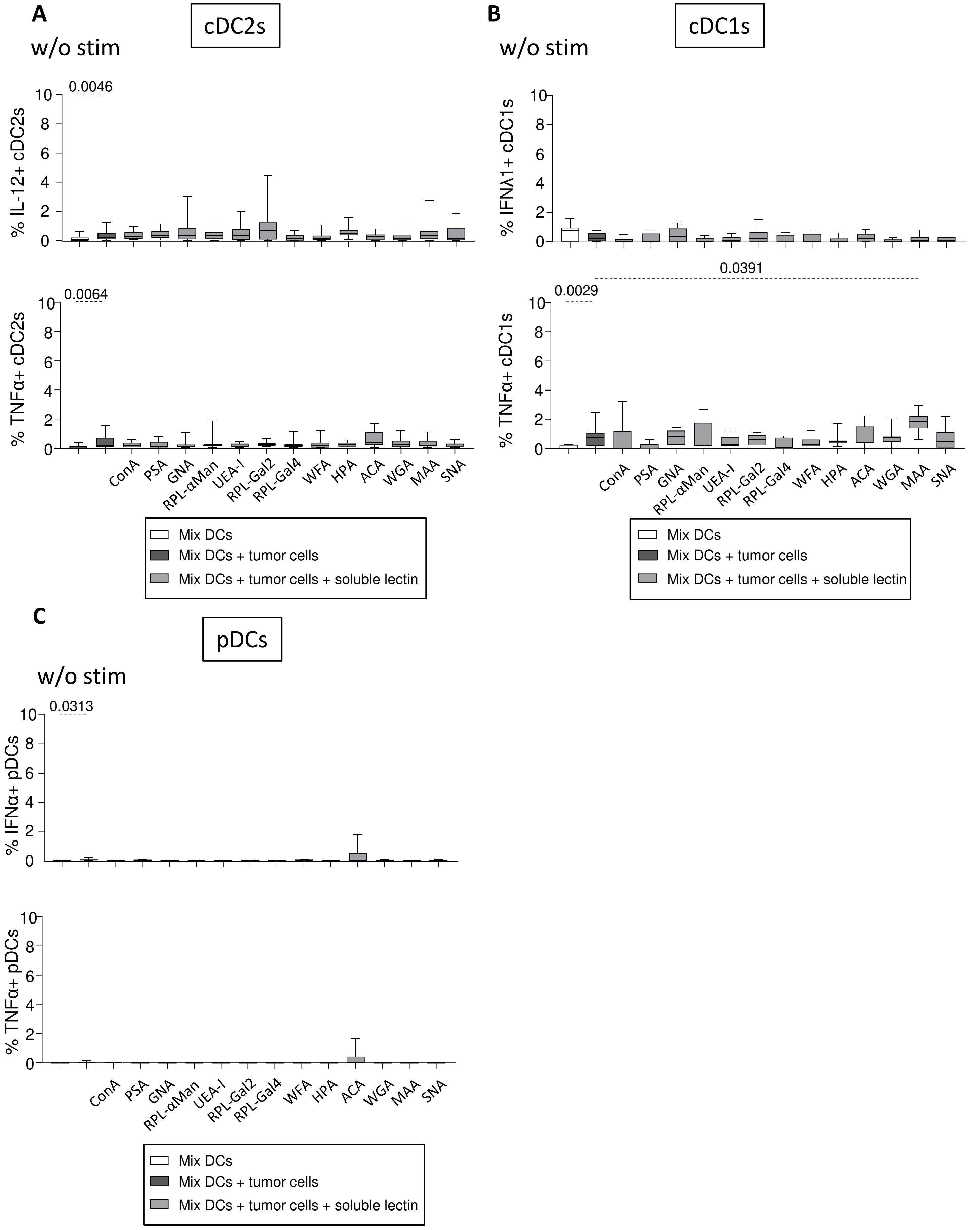

Suppl Figure 12

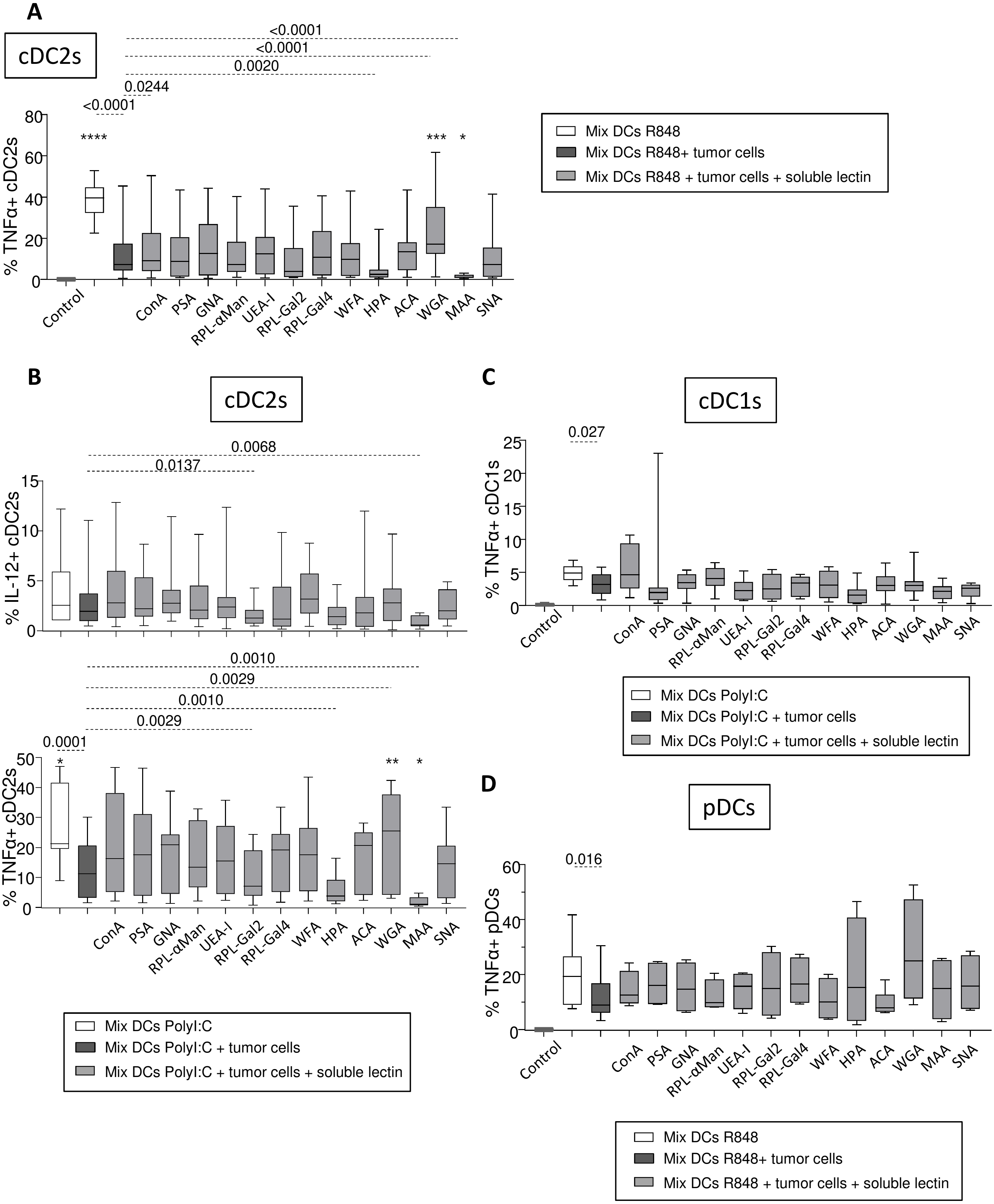

Suppl Figure 13

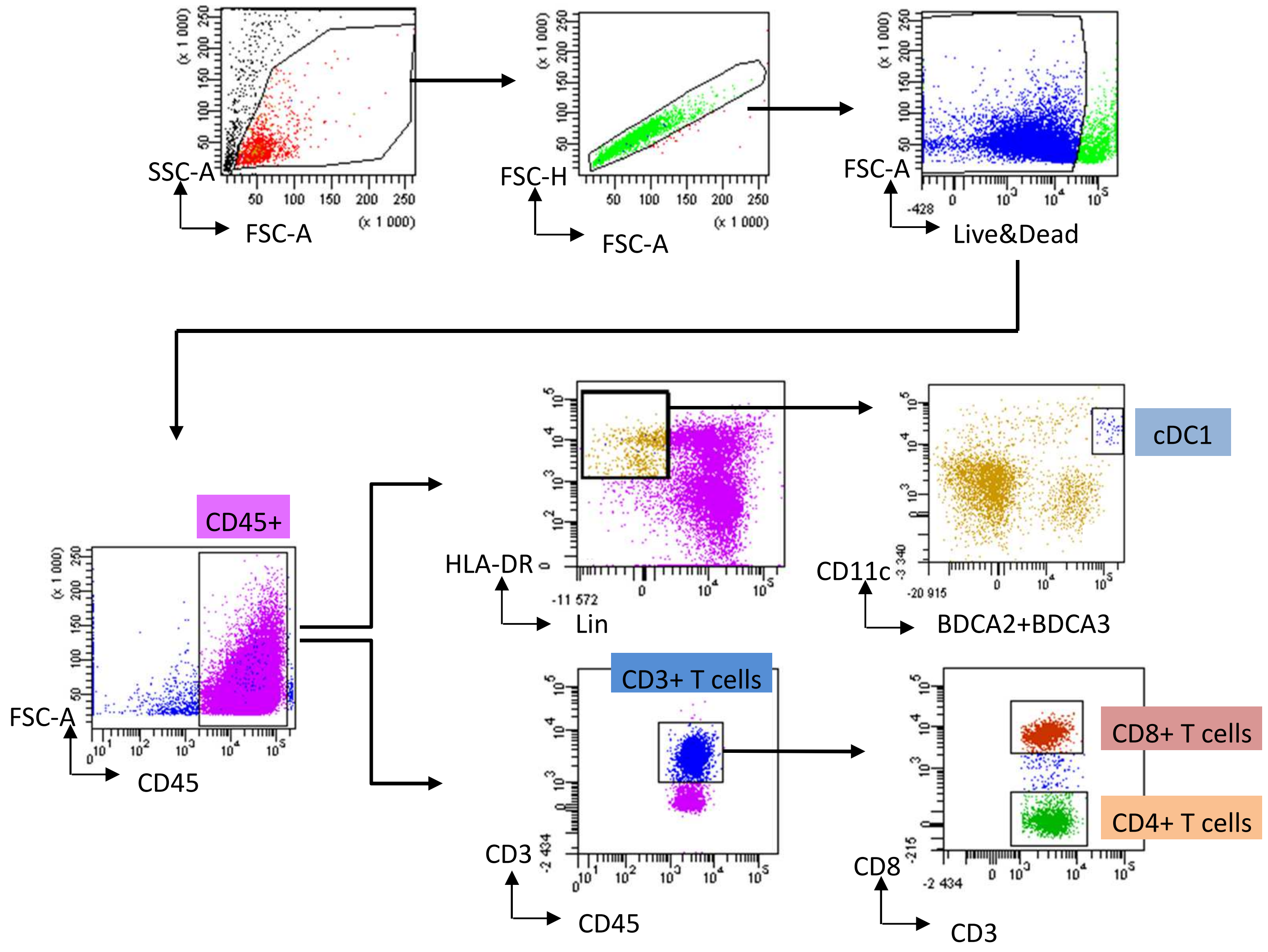

Supplementary table 1: Clinical features of patients from whom tumor cell lines were derived

Patient clinical features									From diagnosis time		From sampling time	
#	sample type	sex	age	Breslow (mm)	Clark	Ulcer	treatment before sampling	TNM classification (at sampling time)	PFS	OS	PFS	OS
1	lymph node metastasis	F	61	4.1	IV	yes	no	IIIc (T4b N1b M0)	2	>129	1	>128
2	sub-cutaneous metastasis	F	ND	2.5	IV	ND	IFNα	ND	70	97		26
3	lymph node metastasis	M	75	3	IV	yes	surgery	IIIc (T3b N3 M0)	7	>64	>46	>56
4	lymph node metastasis	M	59	2.3	IV	no	surgery	IIIc (T3a N3 M0)	138	144	5	6
5	lymph node metastasis	F	35	7.5	IV	ND	surgery ; chemotherapy	IV	42	72		3
6	sub-cutaneous metastasis	M	48	3.5	IV	ND	surgery	IIIc	58	216		153
7	lymph node metastasis	F	43	1.5	III	no	no	III	22	> 84		>146
8	sub-cutaneous metastasis	F	67	1.3	III	no	surgery	IV (T2a N0 M1a)	37	43		3
9	lymph node metastasis	M	39	0.75	IV	no	ND	IIIc	10	34		23
10	lymph node metastasis	M	69	3.5	IV	yes	no	IIIc	10	30	8	28
11	sub-cutaneous metastasis	F	76	5	IV	yes	chemotherapy	IV (T4b N2b M1c)	7	21		3
12	ND	F	72	3.09	IV	yes	no	ND	4	6		2
13	cutaneous metastasis	M	58	2.3	IV	yes	surgery ; chemotherapy	IV	37	58		1
14	lymph node metastasis	M	25	ND	ND	ND	no	ND		31		30
15	lymph node metastasis	F	84	4	IV	yes	no	IIIc	3	48	<44	44
16	ND	F	46	1.4	III	no	no	IV (T2a N1b M1d)	1	11	8	10
17	primary tumor or sub-cutaneous	F	80	ND	ND	ND	no	IIIc (Tx N3 M0)		42		42
18	ND	M	46	1.4	IV	no	no	ND	160	218	36	58
19	lymph node metastasis	M	33	6.9	IV	no	surgery	IV (T4a N3 M1c)	62	74	7	10
20	lymph node metastasis	F	75	1	III	no	surgery	IV (T1a N1b M1a)	69	87	1	16
21	lymph node metastasis	F		ND	ND	ND	ND	IV (Tx N1b Ma1)		44		43
22	lymph node metastasis	M	44	7	IV	yes	surgery	IV	1	12	1	11
23	lymph node metastasis	F	63	5.1	III	yes	ND	IIIc	4	7		3

ND: not determined

<u>Supplementary table 2</u>: Panel of lectins used for GLYcoPROFILES (lectin array performed by GLYcoDiag) and their glycan structures specificity

Short name	Common name	Glycan structures specificities
ACA	Amaranthus Caudatus Agglutinin	Galβ3GalNAca-O-R (TF-antigen)
AIA	Autocarpus Intergrifolia Agglutinin	Galα6 or Galβ(1,3)GalNAc (TF-antigen) >> lactose
BC2L-A	Burkholderia Cenocepacia Lectin A	Dimanoside : Man(α-1,3)Man > Man(α-1,6)Man > Man(α-1,2)Man
ConA	Concanavalin Agglutinin	αMan > αGlc
GNA	Galanthus Nivalis Agglutinin	Terminal αMan, Man(α-1,3)Man
HPA	Helix Pomatia Agglutinin	Terminal αGalNAc
MAA	Maackia Amurensis Agglutinin	Neu5Ac(α2,3)Gal(β1,4)Glc
PNA	Peanut Agglutinin	Lactose, Galβ(1,3)GalNAc (TF-antigen)
PSA	Pisum Sativum Agglutinin	αMan/αGlc > αGlcNAc, a6 fucosylation of the N-linked GlcNAc promotes binding.
RPLGal2	Recombinant Prokaryotic Lectin Galactose 2	Terminal αGal > αGalNAc
RPLGal4	Recombinant Prokaryotic Lectin Galactose 4	Terminal βGal, LacNAc and Lewis x
RPLαMan	Recombinant Prokaryotic Lectin αMannose	Fuc/Man: Lewis a, Lewis x and terminal αMan
SNA	Sambucus Nigra Agglutinin	Neu5Acα(2,6)Gal/GalNAc
UEA-I	Ulex Europaeus Agglutinin	Fucα2Galβ4GlcNac, not inhibited by internal fucose
WFA	Wisteria Floribunda Agglutinin	GalNAc(α1,6)Gal > GalNAc(α1,3)GalNAc (Forssman antigen) > GalNAc
WGA	Wheat Germ Agglutinin	GlcNAc; GlcNAcβ4 oligomers, core of Asn linked oligasacchide; Neu5Ac

Supplementary table 3: Impact of the glyco-code (percentage of lectin fixation) of tumors on patient's clinical outcome (Log rank test analysis).

Log-rank (*P*-values)	ALL TUMORS			
	PFS diagnosis	OS diagnosis	PFS sampling	OS sampling
ConA	0.044	0.044	0.594	0.798
PSA	0.902	0.569	0.594	0.881
GNA*	0.535	0.622	0.460	0.877
BC2LA	0.728	0.763	0.940	0.637
ACA*	0.188	0.162	0.776	0.301
WFA	0.392	0.450	0.140	0.711
HPA	0.122	0.230	0.055	0.980
AIA*	0.817	0.404	0.460	0.123
PNA	0.218	0.864	0.234	0.464
RPL-Gal2	0.742	0.963	0.228	0.211
RPL-Gal4	0.353	0.897	0.630	0.214
WGA	0.929	0.289	0.819	0.231
UEA-I*	0.020	0.025	0.630	0.393
RPL-αMan	0.158	0.964	0.119	0.357
MAA	0.422	0.016	0.820	0.005
SNA	0.211	0.720	0.594	0.030

*separated by 20% fixation

<u>Supplementary table 4:</u> Correlation between tumor glyco-code (percentage of lectin fixation) and immune infiltrate (Spearman correlation).

Spearman correlation (*r*. *P*-value)	Patient tumor infiltrate													
	% CD45$^+$ cells		% CD3$^+$ cells		% pDCs		% cDC2s		% cDC1s		% CD8$^+$ T cells		% CD4$^+$ T cells	
	r	*P*-value	*r*	*P*-value	*r*	*P*-value	*r*	*P*-value	*r*	*P*-value	*r*	*P*-value	*r*	*P*-value
ConA	-0.372	*0.081*	0.289	*0.295*	-0.035	*0.880*	0.033	*0.948*	0.708	*0.018*	0.436	*0.183*	0.445	*0.173*
PSA	-0.154	*0.483*	-0.029	*0.923*	-0.171	*0.459*	-0.617	*0.086*	0.274	*0.412*	0.082	*0.818*	0.409	*0.214*
GNA	-0.047	*0.830*	-0.221	*0.427*	-0.060	*0.796*	-0.367	*0.336*	0.384	*0.243*	0.045	*0.903*	0.291	*0.386*
BC2LA	-0.012	*0.957*	0.232	*0.404*	-0.328	*0.147*	-0.250	*0.521*	0.324	*0.328*	0.345	*0.299*	-0.364	*0.273*
ACA	-0.288	*0.183*	0.311	*0.259*	0.139	*0.549*	0.233	*0.552*	0.219	*0.514*	-0.755	*0.010*	-0.500	*0.122*
WFA	0.073	*0.740*	0.536	*0.042*	-0.290	*0.202*	-0.417	*0.270*	-0.475	*0.142*	0.345	*0.299*	-0.264	*0.435*
HPA	-0.066	*0.770*	0.487	*0.068*	0.010	*0.967*	-0.317	*0.449*	0.438	*0.205*	0.092	*0.795*	-0.376	*0.255*
AIA	-0.147	*0.503*	0.350	*0.201*	0.017	*0.942*	0.000	*1.000*	0.352	*0.287*	-0.245	*0.468*	-0.236	*0.485*
PNA	-0.099	*0.652*	-0.032	*0.914*	-0.351	*0.118*	0.170	*0.668*	0.505	*0.115*	0.432	*0.189*	0.147	*0.669*
RPLGal2	0.026	*0.914*	0.006	*0.993*	0.139	*0.582*	-0.216	*0.636*	0.477	*0.195*	-0.577	*0.110*	-0.084	*0.833*
RPLGal4	0.018	*0.937*	0.040	*0.891*	-0.243	*0.303*	-0.395	*0.332*	0.191	*0.593*	0.615	*0.050*	0.339	*0.307*
WGA	-0.342	*0.110*	0.304	*0.271*	-0.055	*0.812*	0.317	*0.410*	0.808	*0.004*	-0.027	*0.946*	0.100	*0.776*
UEA-I	0.086	*0.717*	-0.599	*0.021*	0.092	*0.716*	-0.393	*0.396*	-0.324	*0.388*	-0.340	*0.333*	0.067	*0.857*
RPL-aMan	-0.004	*0.986*	-0.096	*0.734*	-0.087	*0.707*	-0.217	*0.581*	0.329	*0.321*	-0.045	*0.903*	0.045	*0.903*
MAA	-0.104	*0.638*	0.079	*0.783*	-0.441	*0.045*	-0.217	*0.581*	0.306	*0.357*	0.355	*0.286*	0.309	*0.356*
SNA	-0.200	*0.361*	0.150	*0.593*	0.087	*0.709*	-0.417	*0.270*	0.397	*0.225*	0.336	*0.313*	0.555	*0.082*
